# Analysis of immunoglobulin transcripts and hypermutation following SHIV_AD8_ infection and protein-plus-adjuvant immunization

**DOI:** 10.1038/ncomms7565

**Published:** 2015-04-10

**Authors:** Joseph R. Francica, Zizhang Sheng, Zhenhai Zhang, Yoshiaki Nishimura, Masashi Shingai, Akshaya Ramesh, Brandon F. Keele, Stephen D. Schmidt, Barbara J. Flynn, Sam Darko, Rebecca M. Lynch, Takuya Yamamoto, Rodrigo Matus-Nicodemos, David Wolinsky, NISC Comparative Sequencing Program, Betty Barnabas, Robert Blakesley, Gerry Bouffard, Shelise Brooks, Holly Coleman, Mila Dekhtyar, Michael Gregory, Xiaobin Guan, Jyoti Gupta, Joel Han, Shi-ling Ho, Richelle Legaspi, Quino Maduro, Cathy Masiello, Baishali Maskeri, Jenny McDowell, Casandra Montemayor, James Mullikin, Morgan Park, Nancy Riebow, Karen Schandler, Brian Schmidt, Christina Sison, Mal Stantripop, James Thomas, Pamela Thomas, Meg Vemulapalli, Alice Young, Martha Nason, Nicholas M. Valiante, Padma Malyala, Ennio De Gregorio, Susan W. Barnett, Manmohan Singh, Derek T. O'Hagan, Richard A. Koup, John R. Mascola, Malcolm A. Martin, Thomas B. Kepler, Daniel C. Douek, Lawrence Shapiro, Robert A. Seder

**Affiliations:** 1 Vaccine Research Center, National Institute of Allergy and Infectious Diseases, National Institutes of Health, Bethesda, Maryland 20892, USA; 2 Department of Biochemistry, Columbia University, New York, New York 10032, USA; 3 State Key Laboratory of Organ Failure Research and National Clinical Research Center for Kidney Disease, Nanfang Hospital, Southern Medical University, Guangzhou, Guangdong 510515, China; 4 Laboratory of Molecular Microbiology, National Institute of Allergy and Infectious Diseases, National Institutes of Health, Bethesda, Maryland 20892, USA; 5 Department of Microbiology and Immunology, Boston University, Boston, Massachusetts 02118, USA; 6 AIDS and Cancer Virus Program, Leidos Biomedical Research Inc., Frederick National Laboratory, Frederick, Maryland 21702, USA; 7 Biostatistics Research Branch, National Institute of Allergy and Infectious Diseases, National Institutes of Health, Bethesda, Maryland 20892, USA; 8 Novartis Vaccines and Diagnostics, Cambridge, Massachusetts 02139, USA; 9 NIH Intramural Sequencing Center, National Human Genome Research Institute, Bethesda, Maryland 20852, USA.

## Abstract

Developing predictive animal models to assess how candidate vaccines and infection influence the ontogenies of Envelope (Env)-specific antibodies is critical for the development of an HIV vaccine. Here we use two nonhuman primate models to compare the roles of antigen persistence, diversity and innate immunity. We perform longitudinal analyses of HIV Env-specific B-cell receptor responses to SHIV_AD8_ infection and Env protein vaccination with eight different adjuvants. A subset of the SHIV_AD8_-infected animals with higher viral loads and greater Env diversity show increased neutralization associated with increasing somatic hypermutation (SHM) levels over time. The use of adjuvants results in increased ELISA titres but does not affect the mean SHM levels or CDR H3 lengths. Our study shows how the ontogeny of Env-specific B cells can be tracked, and provides insights into the requirements for developing neutralizing antibodies that should facilitate translation to human vaccine studies.

## Abstract (web summary)

HIV vaccine development will be facilitated by having animal models that are predictive for translation to humans. Here, the authors use two nonhuman primate models to compare the effects of natural infection and different adjuvants on antigen persistence, diversity and humoral immunity.

## Graphical abstract

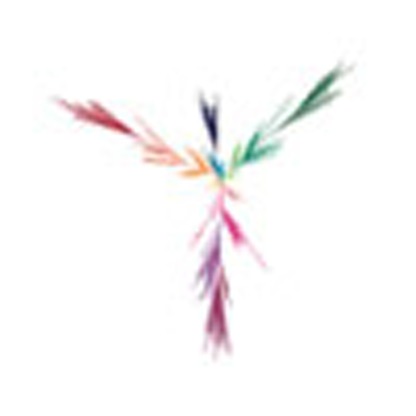

A highly successful preventative HIV vaccine will likely require antibodies that neutralize HIV and block virus acquisition. One of the greatest challenges to HIV vaccine development is the elicitation of antibodies with sufficient breadth and potency to counter the genetic diversity of strains that may establish an infection^1^,^2^. Over the past several years, the cloning and characterization of a number of broadly neutralizing monoclonal antibodies (bNAbs) from HIV-infected humans has identified distinct sites on the HIV Envelope (Env) that are vulnerable to neutralization, and defined several characteristics critical for their protective function^3^,^4^. Many of these bNAbs have high levels of somatic hypermutation (SHM)^5^,^6^,^7^ or long third complementarity-determining regions of the heavy chain (CDR H3)^3^,^8^.

To improve HIV vaccine development, an experimental preclinical animal model is needed to assess how B-cell lineages are elicited and antibodies mature in response to vaccination or infection. Preclinical models using rodents and rabbits may be limited in their ability to elicit responses similar to those characteristic of human bNAbs because of their evolutionarily divergent immunoglobulin (Ig) gene repertoires^9^ or lack of natural CD4 expression. In contrast, nonhuman primates (NHPs) are capable of eliciting cross-reactive neutralizing responses to the V3/glycan site following SHIV_AD8_ infection^10^, tier 1 neutralizing antibodies to the CD4-binding site^11^ and binding antibodies to the membrane proximal external region^12^ following Env protein or peptide vaccination. The high similarity between the antibody genes of humans and NHPs may underlie the ability to elicit such similar responses^9^,^11^. Moreover, because NHPs are most similar to humans in other immunologic aspects, such as tissue-specific Toll-like receptor (TLR) expression^13^,^14^, they provide greater predictability of human vaccine responses than rodent models^15^,^16^.

Recent studies of neutralizing antibody responses in HIV-infected individuals have used next-generation sequencing (NGS) to study the genetic record of antibody development encoded in peripheral memory B cells^17^,^18^,^19^. The germline-encoded antibody segments (V, D and J) provide critical elements for interpreting these data. The currently available heavy chain (H_C_) gene repertoire for rhesus macaque^11^,^20^ was obtained by whole-genome sequencing of a single animal with 5 × depth of coverage. Here we report a new draft database of V_H_ gene sequences from 10 Indian-origin rhesus macaques acquired using Illumina deep sequencing to ∼50–100 × coverage. Using this new draft database, we applied B-cell Ig transcript analysis methods similar to those used previously to interrogate human repertoires^18^,^19^. In this study, longitudinal analyses of two cohorts were performed to address the critical questions of how antigen load, diversity, persistence and innate immunity alter antibody responses: (1) NHP infected with SHIV_AD8_ provide a model with persistent and diverse Env antigens that has been shown to induce potent cross-clade serum-neutralization responses^10^,^21^,^22^; and (2) NHPs vaccinated with gp140 Env protein and eight different adjuvants (alum or MF59 with or without TLR4 or TLR7 ligands, pIC:LC or immune stimulator complexes (ISCOMs)), which were chosen because they are clinically approved or in advanced development, and because they mediate their effects through distinct innate mechanisms that could influence B-cell immunity. From both of these studies, peripheral antibody transcripts isolated from Env-specific B cells were sequenced to assess SHM, CDR H3 length and variable heavy (V_H_) gene repertoire.

The data presented here using the NHP vaccine model confirm that a number of clinically based adjuvants are effective for enhancing the magnitude of antibody response but, remarkably, are not able to increase SHM. Alternatively, during chronic SHIV_AD8_ infection, antigen diversity and persistence appear critical for enhancing the potency and breadth of neutralizing Env antibody responses and SHM.

## Results

### Development of an NGS platform for Env-specific B cells

To study B-cell ontogeny and characterize Ig maturation features, an NGS platform was developed that could be used to study a large number of NHPs (Supplementary Fig. 1). Briefly, Env probe-specific B cells were bulk-sorted from individual animals at various time points after SHIV_AD8_ infection or Env and adjuvant vaccination (Supplementary Fig. 2), IgG H_C_ transcripts were amplified by multiplexed primer PCR with unique barcodes, and sequenced by 454 pyrosequencing. Raw reads were then filtered for quality and redundancy and mapped to a newly generated rhesus macaque Ig draft reference database (new accession codes KP710506 to KP710583 and NW_001121239 to NW_001121240 from previous IMGT database). The draft database comprises 58 V_H_ genes, with 98 alleles in total (Supplementary Table 1). There were 9, 3, 28, 12, 3, 1 and 2 members of the VH1, VH2, VH3, VH4, VH5, VH6 and VH7 families, respectively. Temporary names for these genes were assigned; the closest mapping between NHP and human V_H_ genes^23^, as well as to a previously published NHP database^20^, is shown in Supplementary Fig. 3.

To obtain a detailed understanding of B-cell responses to infection and vaccination, three major analyses were performed on the unique sequences obtained from NGS: (1) SHM, calculated as percent divergence from germline at the nucleotide (nt) level; (2) CDR H3 length; and (3) the V_H_ gene origin. Unique sequences were used for each of these analyses because, for small sequence differences, true somatic variants cannot be distinguished from PCR-induced replicates and redundant reads could affect gene-usage distributions. The inclusion of redundant sequences yielded very similar results for the mean divergence analyses compared with using only unique reads (Supplementary Fig. 4a). To further ensure that gene-usage distributions obtained with multiplex primer PCR were not biased, rapid amplification of cDNA end (RACE) PCR, which uses a common 5′ primer to avoid primer-associated amplification bias, was used for comparative purposes. Similar results were obtained by these different methods (Supplementary Fig. 5) demonstrating that the multiplex primer PCR approach did not significantly change the V_H_ gene distribution.

### Viral load and Env sequence diversity during SHIV_AD8_ infection

**Figure 1:**
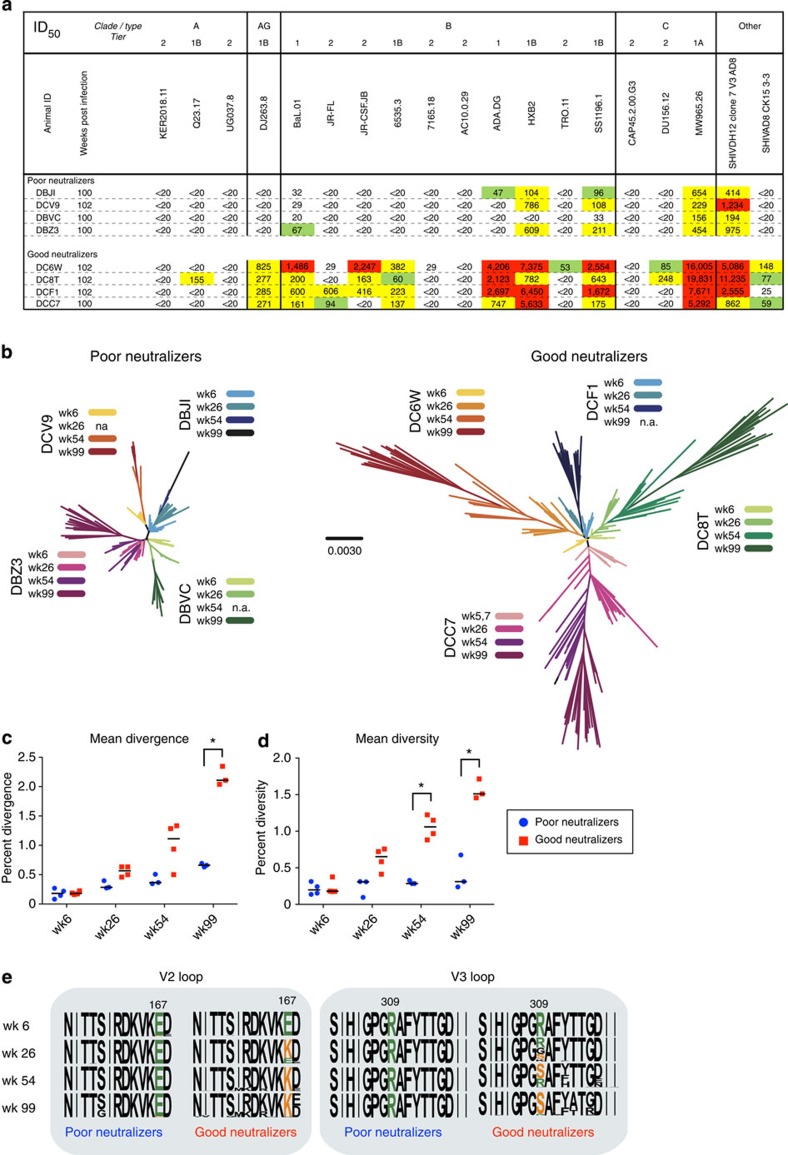
Serum neutralization breadth and viral diversity during SHIV_AD8_ infection. (**a**) Plasma neutralization of selected viruses 100–102 weeks post infection with SHIV_AD8_. Eight animals were segregated into good and poor neutralizers on the basis of the potency and breadth of their responses. ID_50_ values are shown; colours indicate potency: 40–99, green; 100–999, yellow; ≥1,000, red. (**b**–**d**) Viral sequencing was performed on plasma from SHIV-infected NHP at week 6, 26, 54 and 99, except where indicated. (**b**) Phylogenic trees of Env sequences rooted to the inoculum sequence for good and poor neutralizers. Colours indicate time points; scale bar indicates 0.3% divergence. The mean divergence (**c**) or diversity (**d**) was calculated for each animal at the indicated time points. Horizontal bars indicate group medians; *, significant discovery by multiple *t*-test comparison. Sequence data were not available for DBVC wk 54, DCV9 wk 26 and DCF1 wk99 because of low viral titres. (**e**) Consensus Env sequences at the indicated time points highlighting the V2 and V3 loops. Conserved mutations at amino-acid positions 167 and 309 are shown (orange) compared with the original residue (green) in the good and poor neutralizer animals.

To study the development of neutralizing antibodies, NHPs were infected with SHIV_AD8_, a tier 2B virus that has been shown to induce potent cross-reactive serum neutralization in NHP^10^,^21^,^22^. This model shows features of HIV infection in humans such as continuous CD4^+^ T-cell loss, sustained plasma viraemia and clinical immunodeficiency. Plasma from infected NHPs was screened over the course of infection for neutralization against a diverse panel of tier 1 and 2 viruses. Animals were segregated based the basis of neutralization breadth and potency: four were categorized as ‘good neutralizers' and four as ‘poor neutralizers' (Fig. 1a). Poor neutralizers were categorized as demonstrating limited neutralization of tier 1A and 1B strains, while good neutralizers were those that showed much stronger tier 1 neutralization as well as moderate activity against several tier 2 strains, including the autologous Env, CK15 3-3. Generally, neutralization developed after 50 weeks post infection^10^,^21^ (Supplementary Fig. 6a).

To determine what factors may influence the development of neutralization in the SHIV_AD8_ model, viral load and diversity were assessed during infection. Longitudinal analysis showed that good neutralizers maintained viral loads 1–2 orders of magnitude higher, and had a more rapid decline in peripheral CD4 counts, than did poor neutralizers (Supplementary Fig. 6b,c). To track viral evolution and diversity, single-genome amplification (SGA) of blood plasma virus was performed over the course of the infection (Fig. 1b). In total, 441 single genomes were amplified with a mean of 55 per animal. Notably, Env sequences in the blood from good neutralizers were significantly more divergent from the infecting inoculum (*P*<0.0001, multiple *t*-tests, Fig. 1c) and showed greater sequence diversity over time than virus from poor neutralizers (*P*=0.002, multiple *t*-tests, Fig. 1d). Virus in good neutralizer animals evolved about four times faster than in poor neutralizers (0.02 versus 0.005 substitutions per site per year) over a period of 100 weeks. Of note, E to K mutations at position 167 in the V2 loop epitope and R to S mutations at position 309 in the V3 loop epitope were consistently found in virus from all of the good neutralizers but not the poor neutralizers (Fig. 1e). These conserved mutations were first observed at week 26 and became predominant in the population over time, suggesting immunological pressure at these sites. Taken together, these data highlight the dynamic interactions between virus and neutralization in the macaque SHIV_AD8_ model and provide an opportunity to study how these virologic characteristics relate to B-cell Ig sequences.

### SHIV_AD8_ infection analysis of SHM and neutralization

**Figure 2:**
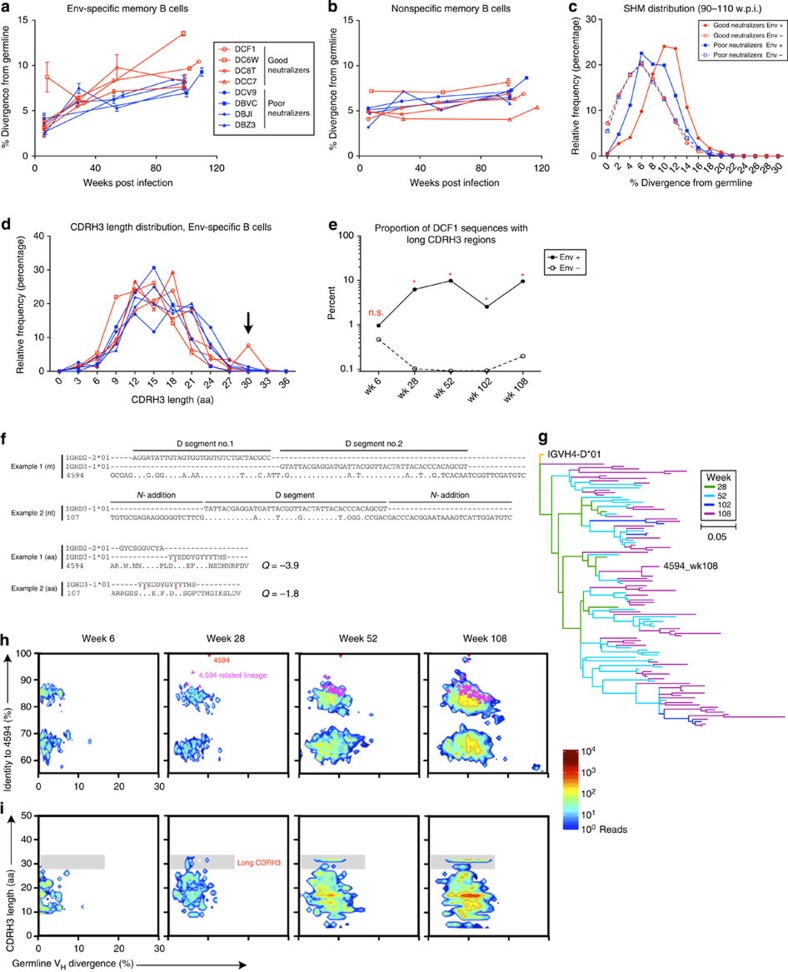
Next-generation sequencing of antigen-specific B cells after SHIV_AD8_ infection. Data are shown from four good neutralizers, red; and four poor neutralizers, blue. (**a**,**b**) SHM for each animal over time; each symbol represents the average percent divergence from germline for Env-specific (gp120+) (**a**) or nonspecific (gp120−) (**b**) sequences from a given animal; error bars indicate s.e.m. (**c**) Histogram representation of SHM distribution from all sequences from 90–110 weeks post infection; binning averaged in 2% increments. (**d**) CDR H3 length distribution of Env-specific sequences from individual animals for all time points combined. Colour/symbol scheme as in **a**; binning averaged in 3-aa increments; arrow indicates population of sequences with long CDR H3 regions. (**e**) Proportion of unique sequences with long CDR H3 regions from DCF1 at the indicated time points. *χ*^2^-test with Yates correction was used to calculate *P* values between proportions from Env+ or − samples at each time point; n.s., not significant; *P<0.0001. (**f**) Long CDR H3 regions from DCF1 may be derived in at least two unique ways. Example 1 depicts the CDR H3 region of sequence 4594, arising from V(DD)J recombination. Example 2 depicts the CDR H3 region of sequence 107, arising from *N-*addition. Bold text indicates mature antibody sequence; red stars indicate predicted tyrosine sulfation; CDR H3 charge is shown. (**g**) Divergence over time of long CDR H3 antibodies from the parent lineage that includes sequence 4594. A phylogenic tree was constructed by maximum likelihood and rooted to the IGHV4D*01 allele and is colour-coded by time point. (**h**,**i**) Two-dimensional plots depicting Env-specific sequences from animal DCF1 at the indicated time points. Sequences are plotted by their V_H_ divergence from germline and their identity to the 4594 H_C_ (**h**); or by their CDR H3 length (**i**). Red triangle indicates sequence 4594; magenta triangles indicate sequences related to 4594; shaded boxes indicate reads with long CDR H3 regions.

To determine how SHIV_AD8_ infection influences Ig maturation, AD8 gp120 Env-specific and nonspecific memory B cells from infected animals were isolated at various time points post infection, sequenced and analysed (Supplementary Table 2). A total of 5,526 unique H_C_ sequences were derived from 28,000 Env-specific cells, and 311,274 H_C_ sequences were derived from ∼1,313,000 nonspecific cells. Env-specific B cells accumulated mutations over the course of infection and two of the four good neutralizers had higher divergence levels than the poor neutralizers (Fig. 2a). As a control, sequences from nonspecific memory B cells did not significantly accumulate mutations over the course of the infection (Fig. 2b). Indeed, by 110 weeks post infection Env-specific B cells had accumulated more mutations from germline than had nonspecific cells (Fig. 2c). These data show that SHIV_AD8_ infection results in the accumulation of SHM that is HIV-specific and correlates with serum neutralization.

### Tracking CDR H3 length during SHIV_AD8_ infection

Long CDR H3s are another defining characteristic of several human HIV bNAbs. Similar to what has been observed in humans^24^, nonspecific memory B cells from SHIV_AD8_-infected animals had a median CDR H3 length of 14 aa, and Env-specific memory B cells had a median length of 16 aa. One of the good neutralizer animals, DCF1, had a large population of H_C_ sequences with CDR H3s of 31 aa in length (Fig. 2d), which gradually increased in proportion over time (Fig. 2e). The ontogeny of these sequences was further analysed to assess potential mechanisms of how long CDR H3 regions develop. Most of the long CDR H3 regions from this animal, such as in sequence no. 107, attained their length solely through nucleotide (*N-*) addition; however some of the CDR H3s, such as in sequence no. 4594, were generated through V(DD)J recombination (Fig. 2f). All of these CDR H3 regions are anionic and are all predicted to have one or more sulfated tyrosines, similar to human V1V2-targeted bNAbs^25^,^26^. These long CDR H3 antibodies comprised 14 lineages with CDR H3 lengths ranging from 28 to 31 aa, the largest of which includes sequence no. 4594 (Fig. 2g). In all, nine of the fourteen long CDR H3 lineages were first observed at week 28 and three of these lineages grew in proportion and diversity over time (Fig. 2h), although the average CDR H3 length remained constant (Fig. 2i). These data demonstrate that CDR H3 length is generated primarily during recombination and show convergent evolution from separately generated lineages over the course of infection.

### Influence of adjuvants on antibody titre and breadth

Chronic SHIV_AD8_ infection results in persistent innate activation and fluctuations in the type and number of CD4^+^ T cells, which may be critical for the induction of cross-reactive serum neutralization. Therefore, we assessed how adjuvants that mediate their effects through different innate immune pathways would influence B-cell maturation in the context of Env protein vaccination. MF59 has been shown to recruit neutrophils, increase antigen uptake and increase antibody affinity^27^,^28^. TLR4 agonists are the first TLR ligands approved for use in humans and activate certain dendritic cell subsets and macrophages leading to potent antibody responses^29^. Agonists to TLR3 (pIC:LC) and TLR7 were used because they induce robust production of interleukin (IL)-12 and Type I interferon (IFN), leading to potent CD4^+^ T_H_1 immunity and antibody production^14^; TLR7 is also expressed in B cells, allowing for direct adjuvant activation. ISCOMs were also tested, as they have also been shown to be potent stimulators of both T- and B-cell immunity^30^. Alum was used a benchmark adjuvant based on its longstanding clinical use for enhancing antibody responses.

**Figure 3:**
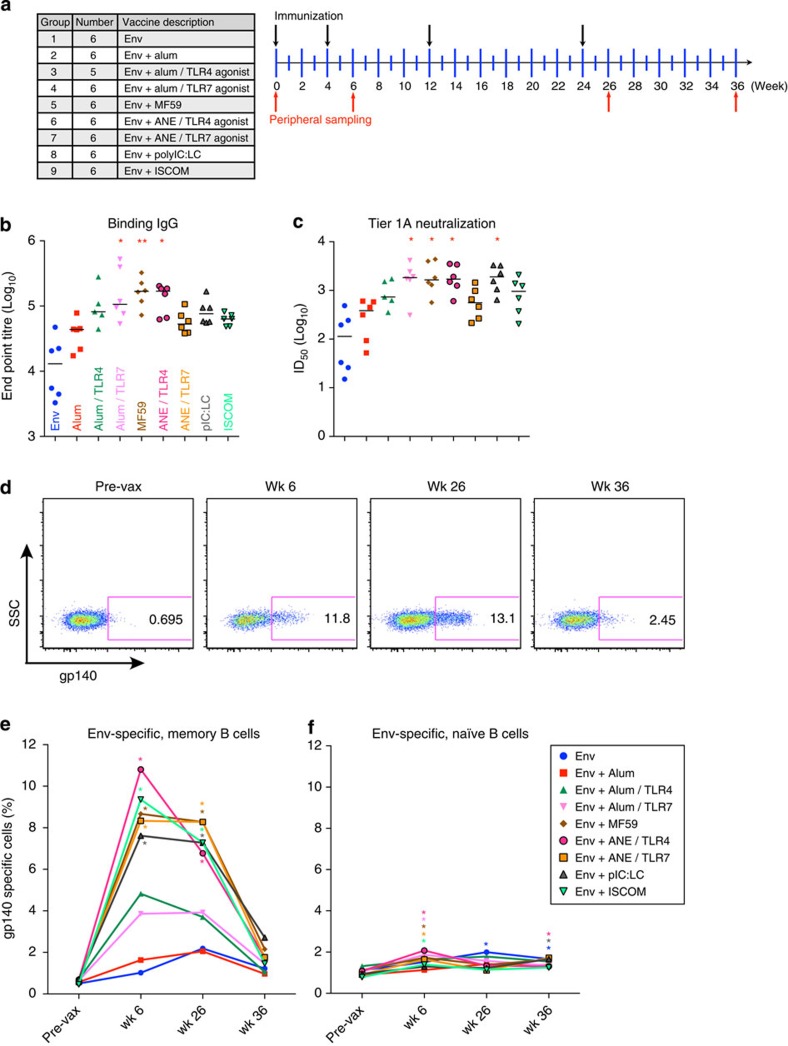
Vaccination with adjuvants results differential effects on humoral and B cellular responses. (**a**) Vaccination project overview. Nine vaccines were given to 53 NHP in a homologous prime-boost manner at 0, 4, 12 and 24 weeks (black arrows). PBMC sampling was performed before vaccination (pre-vax) and 6, 26 or 36 weeks after the prime (red arrows). (**b**) Env-specific IgG-binding titres at week 26. (**c**) Plasma neutralization of tier 1A MW965.26 Env-bearing pseudovirus at week 26. Horizontal bars indicate medians. *P<0.05; **P<0.01 compared with Env+alum group by the Kruskal–Wallis test. (**d**–**f**) Antigen-specific B cells were identified from PBMCs at the indicated time points by binding to a gp140 protein probe followed by flow cytometry. (**d**) Representative flow cytometry plots of antigen-specific memory B cells at the indicated time points after prime. Antigen-specific memory (**e**) or naive (**f**) cells were enumerated at the indicated time points. Data points represent vaccine group means and are depicted as a percent of all IgG^+^ cells. *P<0.05 compared with pre-time point by two-way analyses of variance.

Rhesus macaques (*n*=6 per group) were immunized intramuscularly at 0, 4, 12 and 24 weeks with gp140 TV1ΔV2 Env protein alone, or formulated with alum or MF59 with or without agonists of TLR 4 or 7, or with pIC:LC or ISCOMs (Fig. 3a). It should be noted that unlike the clade B AD8 strain used in the SHIV_AD8_ infection, the clade C Envelope TV1 was used for vaccination. TV1 was used because Novartis had clinical formulations of the protein and adjuvants, thus facilitating the translation of preclinical study findings to humans. Clade C strains are most common in Africa and Asia; therefore, this strain is relevant to these regions where several vaccine trials are currently being planned. Formulations derived from MF59 to include TLR agonists are referred to as adjuvant nano-emulsions (ANE).

To first show how the adjuvants affected humoral immunity, Env-binding titres were assessed from the peak of the response after the final immunization, week 26. Animals receiving alum/TLR7, MF59 and ANE/TLR4 produced the highest IgG titres, while alum/TLR4, pIC:LC and ISCOMs showed a trend towards enhancement over the benchmark adjuvant, alum (Fig. 3b). Plasma neutralization was observed against a panel of seven tier 1 viruses, and the potency among adjuvant groups was similar to the binding titres; activity against tier 2 viruses was largely undetected (Fig. 3c). These results provide an extensive comparison of existing and novel clinically based adjuvant formulations, confirming their ability to increase the magnitude of antibody responses.

### The frequency of Env-specific B cells is modulated by adjuvants

To assess how adjuvants affect Env-specific B cells, these were first quantified from peripheral blood mononuclear cells (PBMCs) by their binding of a labelled gp140 TV1ΔV2 Env probe throughout the course of vaccination (Fig. 3d; Supplementary Fig. 2b). Animals receiving Env alone or Env with alum showed a low frequency of Env-specific B cells, and were increased by addition of the TLR 4 or 7 agonists (Fig. 3e). The MF59, pIC:LC and ISCOM formulations all induced a striking increase in Env-specific cells after two or four immunizations, although these populations significantly contracted by week 36 (Fig. 3e). In contrast, Env-specific naive B cells were found at a much lower frequency throughout the course of vaccination (Fig. 3f).

Env-specific memory B cells were then sorted from 51 of the vaccinated animals after the fourth immunization and processed through the NGS pipeline, yielding 53,563 Ig H_C_ unique sequences from ∼201,000 sorted Env-specific B cells (Supplementary Table 3). These data were subsequently analysed on a per-animal or a per-vaccine basis; each vaccine group has at least four animals and a minimum of 2,000 unique sequences, allowing for robust comparisons at both levels (Supplementary Table 4).

### Adjuvants do not alter overall SHM levels

**Figure 4:**
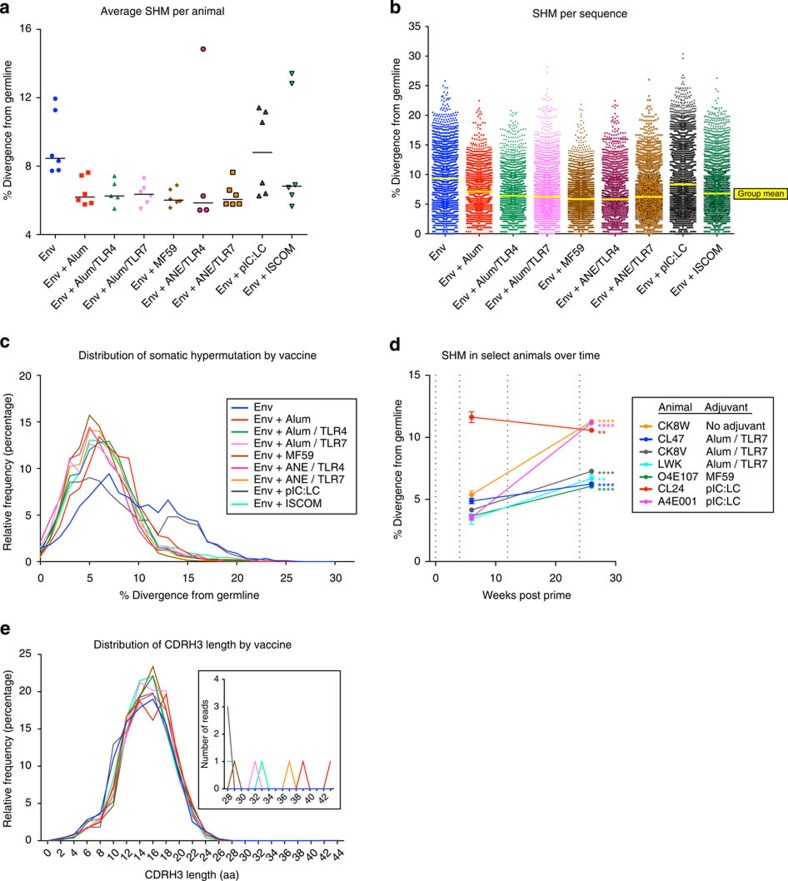
Next-generation sequencing of antigen-specific B cells after protein and adjuvant vaccination. (**a**) SHM for each animal (*n*=51) at week 26; each symbol represents the average percent divergence from germline for sequences from a given animal. Horizontal bars indicate the medians. (**b**) SHM for each vaccine at week 26; each symbol represents the percent divergence from germline for a unique sequence. Horizontal yellow bars indicate the geometric means. (**c**) Histogram representation of SHM at week 26. The distribution is shown as a composite for all unique sequences from a given vaccine; binning averaged in 2% increments. (**d**) Effect of boosting on SHM. Percent divergence of antigen-specific cells sorted at week 6 or 26 is depicted for seven animals. Data points represent the mean SHM for each animal; error bars show s.e.'s; vertical dashed lines represent immunization points; n.s., not significant; **P<0.01; ****P<0.0001 compared with week 6 time point by two-way ANOVA. (**e**) CDR H3 length distribution as a composite from all sequences from a given vaccine; binning averaged in 2-aa increments. Inset highlights the low frequency of reads with long (≥28 aa) CDR H3 regions. Colours denote vaccines as in **c**.

For each V_H_ sequence, CDR and framework regions (FRs) were delineated following IMGT definitions^31^ and the level of SHM within each region was assessed (Supplementary Fig. 7a). Mutations were significantly higher in the CDR1 and CDR2 regions (12%, 15% divergence from germline), compared with the FRs (4–6% divergence). However, the adjuvants did not affect the accumulation of mutations in these regions, nor did they affect the proportion of V_H_ gene families (Supplementary Fig. 7b). The influence of adjuvants on accumulated SHM levels of Env-specific B cells was next determined. The mean divergence from germline after four immunizations (week 26) was analysed for each animal (Fig. 4a) or by grouping all the individual sequences from each vaccine (Fig. 4b,c). Notably, animals immunized with the Env protein alone induced the highest average divergence, while the addition of MF59 (*P*=0.007, mixed effect linear regression model) and ANE/TLR7 (*P*=0.007) had statistically lower levels. The finding that these adjuvants did not increase SHM was subsequently confirmed in an independent follow-up experiment in which SHM levels were assessed after two or three homologous immunizations with several of the same adjuvants (Supplementary Fig. 8a,b). To extend the analysis, we determined whether germline divergence increased after successive immunizations. Env-specific B cells were sequenced from seven animals of different vaccine groups after two immunizations (week 6). Six of these animals showed an increase from week 6 to week 26 (Fig. 4d), showing that successive boosting did increase accumulated SHM for antigen-specific transcripts (Supplementary Fig. 8c).

Although the overall mean divergence levels was not increased with these adjuvants, the germline divergence of particular V_H_ genes was highly variable among the vaccine groups. Using data from the two NHP protein-plus-adjuvant vaccination studies, we found that after mapping sequences to each of the 58 V_H_ genes, some such as *IGHV4L* were largely unaffected by the adjuvant used. In contrast, others such as *IGHV3Q* showed large variations among the adjuvants (Supplementary Fig. 8e). These data suggest the ability of adjuvants to differentially promote the development of B cells with specific V_H_ genes, although further studies should be performed to investigate this phenomenon.

### Adjuvants do not alter the development of CDR H3 length

To conclude the analysis of how adjuvants influence B-cell development, the CDR H3 length distribution for each vaccine group were assessed. The median CDR H3 length was 14 or 15 aa (Fig. 4e; Supplementary Table 4) and none of the vaccine adjuvants elicited a population of Env-specific B cells with long CDR H3 regions; only 11 sequences were found to have a CDR H3 ≥28 aa in length (Fig. 4e, Supplementary Fig. 8d). This finding is consistent with the frequency of long CDR H3 sequences from the naive repertoire (∼0.03%), which was measured by sorting naive B cells from two NHPs before vaccination (Supplementary Fig. 4b).

### Highly mutated Env antibodies arise from specific V_H_ genes

**Figure 5:**
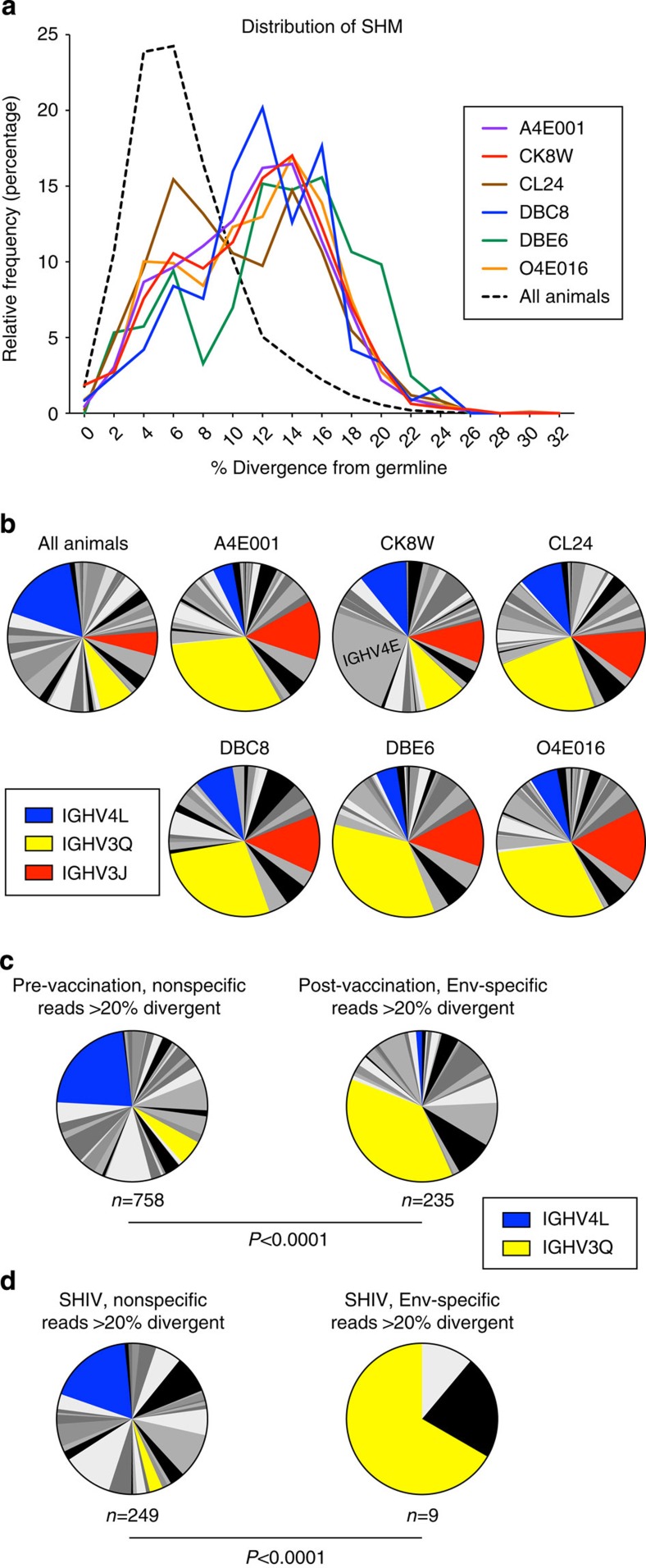
Characteristics of animals and sequences with high levels of SHM at week 26 after vaccination. (**a**) Histogram representation of SHM distribution from animals with an average >10% divergence from germline, compared with all vaccinated animals. Binning averaged in 2% increments. (**b**) V_H_ gene composition for sequences from animals with high SHM compared with the whole data set, all animals. The frequency of reads mapping to each V_H_ gene are represented as a fraction of the total sequences. (**c**) V_H_ gene composition for sequences >20% divergent from germline from a subset of samples taken pre-vaccination (bulk IgG reads) or from Env-specific B cells sorted from all animals post-vaccination. (**d**) V_H_ gene composition for sequences >20% divergent from germline from sorted Env-specific (gp120+) or nonspecific (gp120−) B cells from SHIV-infected animals. *P* values are derived from the Fisher's exact test for the proportion of *IGHV3Q*. The number of sequences in each population subset is indicated under the corresponding pie chart. Sequences mapping to *IGHV4L* (blue), *IGHV3Q* (yellow) and *IGHV3J* (red) are highlighted.

Because some HIV bNAbs accumulate high levels of SHM, the data set was examined for highly mutated antibodies. While the mean divergences from germline genes were similar between the vaccine groups, eight animals from various vaccine groups had high mean divergence levels (Fig. 4a). Furthermore, in all vaccine groups there were animals containing highly divergent sequences (Fig. 4b). Therefore, animals with >10% mean SHM (*n*=6, excluding O4E064 and T5321, which had very low numbers of unique reads) were further assessed for SHM distribution and V_H_ gene composition. These animals had a relatively uniformly higher distribution of mutated sequences compared with all vaccinated animals (Fig. 5a). Of note, they showed an increase in the proportion of two V_H_ genes, *IGHV3Q* and *IGHV3J*, and a decrease in other V_H_ genes, especially *IGHV4L*, which was the most prominent V_H_ gene for all other animals (Fig. 5b). Overall, these data show that there were significant alterations in V_H_ gene usage in animals with the highest levels of SHM.

We next analysed all sequences that were >20% divergent in the Env vaccination study. *IGHV3Q* was again the predominant V_H_ gene, comprising 37% of such reads, with a relatively small frequency of *IGHV4L* (Fig. 5c). As a control, we compared this distribution to reads that were >20% divergent from nonspecific memory B cells (from pre-vaccination samples, *n*=584,634). Here *IGHV4L* was the predominant V_H_ gene used, and *IGHV3Q* comprised only 5% (Fig. 5c). To see whether the preference of high SHM reads for *IGHV3Q* was limited to vaccinated NHP, we analysed highly divergent reads from the SHIV_AD8_-infected animals. Only nine unique highly divergent reads were found, yet six of these mapped to *IGHV3Q* (Fig. 5d). Among highly mutated reads from nonspecific B cells, *IGHV4L* was the most prominent V_H_ gene, as was observed with nonspecific reads from the Env vaccination study. Taken together, these data suggest that high levels of SHM in Env-specific antibodies are associated with a preference for the *IGHV3Q* gene. This finding was confirmed in a follow-up Env protein-plus-adjuvant vaccination study (Supplementary Fig. 8f).

### Preferential V_H_ gene usage following SHIV_AD8_ infection

**Figure 6:**
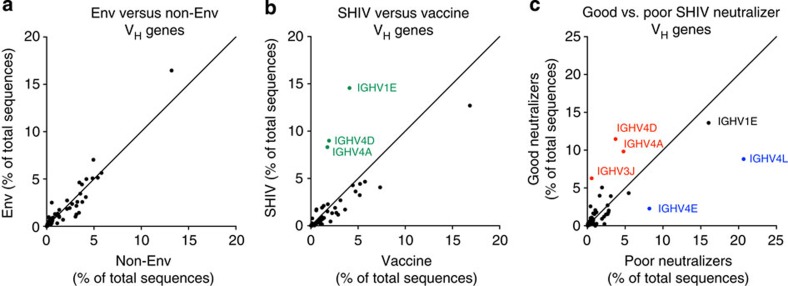
V_H_ gene correlations between Env/adjuvant vaccination and SHIV_AD8_ infection models. The composition of individual V_H_ genes is graphed as the percentage of sequences mapping to each V_H_ gene within each data set. Each dot represents an individual V_H_ gene; diagonal lines indicate the position of genes lacking a preference between data sets. (**a**) HIV Env-specific compared with nonspecific sequences combined from SHIV_AD8_ infection and Env/adjuvant vaccination data sets. (**b**) Env-specific sequences from the SHIV data set compared with the Env/adjuvant vaccination data set. V_H_ genes with enriched composition in the SHIV data set are shown in green. (**c**) Env-specific sequences from SHIV_AD8_ good neutralizers compared with poor neutralizers. V_H_ genes with enriched composition in the good neutralizer data set are shown in red; those enriched in the poor neutralizer data set are shown in blue.

To address whether certain V_H_ genes are preferentially used for Env reactivity or neutralization, a composite analysis of data obtained from both the Env vaccination and SHIV_AD8_ infection studies was performed. Pre-vaccination IgG sequences and nonspecific memory B cells from the SHIV_AD8_ infection study provided a ‘non-Env' data set (*n*=890,460). Env-specific reads from B cells sorted after vaccination and SHIV_AD8_ infection were combined for an ‘Env-specific' data set (*n*=58,942). The proportions of V_H_ genes between these data sets were compared and an enrichment of specific V_H_ genes was assessed. However, there was no preferential V_H_ gene usage between the sequences from the Env- and non-Env-specific data sets (Fig. 6a).

The V_H_ gene composition of Env-specific sequences between the Env vaccination and the SHIV_AD8_ infection studies were then compared. In SHIV_AD8_-infected animals, sequences preferentially mapped to *IGHV1E* (*P*<0.0001, *χ*^2^-test), *4A* (*P*<0.0001) and *4D* (*P*<0.0001), which could represent a ‘V_H_ gene signature' of SHIV_AD8_ infection (Fig. 6b). Furthermore, in comparing V_H_ gene composition between good and poor neutralizers from SHIV_AD8_-infected animals, *IGHV4A* (*P*<0.0001, *χ*^2^-test), *4D* (*P*<0.0001) and *3J* (*P*<0.0001) were more prevalent in the good neutralizers at a high level, while *IGHV4E* (*P*<0.0001) and *4L* (*P*<0.0001) were more prevalent in the poor neutralizers (Fig. 6c). These findings were substantiated by performing the same analysis using Sanger sequenced, single-cell sorted Env-specific B cells from a separate study of SHIV_AD8_-infected NHP (Supplementary Fig. 9). This analysis shows how the NHP model can be used to evaluate which immunoglobulin V_H_ genes play a role in specific immune responses.

## Discussion

Using a high-throughput NGS platform with a newly generated draft Rhesus macaque Ig H_C_ reference database, Env-specific antibody responses were assessed following SHIV_AD8_ infection and Env protein-plus-adjuvant vaccination to gain insight into factors that influence SHM and other characteristics associated with neutralizing antibodies. The SHIV_AD8_ infection model provides a striking contrast in the amount, duration, diversity and conformation of Env antigen compared with homologous Env protein immunization. Nevertheless, it was notable that antigen-specific B cells in both SHIV_AD8_-infected and Env-vaccinated animals had similar mean germline divergence levels at both week 6 and week 26 (Figs 2a and 4d). As the SHIV_AD8_ infection progressed past week 50, animals that maintained their viral loads had increased viral diversity and increased antibody germline divergence, which was associated with the development of cross-reactive neutralization. These data are consistent with several studies of HIV-infected individuals in whom increased viral loads and viral Env diversity were correlated with serum neutralization capacity^32^,^33^,^34^. By contrast, and consistent with other gp140 vaccination studies^35^,^36^, Env-vaccinated NHP did not develop significant cross-reactive neutralization responses. Of note, sequences greater than 20% divergent from germline (which is in the range of many characterized bNAbs^5^) were detected from some animals in all vaccine groups after four immunizations. These data show that levels of SHM accumulated following infection or vaccination may be necessary but are not sufficient to generate potent cross-reactive neutralization.

A major question addressed in this study was whether various adjuvant formulations differed in their capacity to increase SHM levels. Here clinically approved adjuvants (alum, MF59) were evaluated individually as benchmarks or combined with TLR ligands such as TLR 4 and 7 to enhance their potency. These ligands activate innate immune cells such as macrophages and specific DC subsets to secrete IL-6 and Type I IFN, respectively, mediators that can induce B-cell activation and enhance antibody production^37^,^38^,^39^. TLR7 is also expressed in B cells and can directly induce B-cell activation. Moreover, TLR stimulation has been shown to activate AID^40^ and SHM^41^ *in vitro*, and other reports have showed affinity maturation using MF59 in an influenza vaccine^28^ or CpGs in a hepatitis B vaccine^42^, although direct links between affinity and SHM were not explored. Remarkably, we found limited differences in the mean levels of SHM levels induced by the different adjuvants compared with the Env protein alone after four homologous immunizations. We note that for Env-specific B-cell isolation, trimeric gp140 was used in the vaccine adjuvant study and gp120 Env protein used in the SHIV_AD8_ infection study. Both of these proteins are uncleaved molecules, and thus are unable to bind quaternary-specific epitopes and potentially react with non-native epitopes. For future studies, cleaved, stable Env trimers will make better probes for isolating Env-specific B cells^43^.

The correlation over time between viral diversity and the development of neutralization breadth and potency in the SHIV_AD8_ model raises the question of how vaccines might be better designed to induce such a response. The SHIV_AD8_ data (Supplementary Fig. 6a) indicate that some tier 1A reactivity develops before tier 2 neutralization in the good neutralizers. This suggests not that tier 1 antibodies are precursors to tier 2 antibodies but rather that tier 2 antibodies may take longer to develop. The virological differences between good and poor neutralizers do not simply result in improved tier 1 neutralization, but also give rise to greater breadth of neutralization, including tier 2 isolates. Therefore, vaccine regimens mimicking the antigen load and diversity seen during SHIV_AD8_ infection might induce better quality neutralization responses than what is currently observed after homologous immunization. To this end, strategies using sequential or concurrent immunization of multiple diverse Env proteins could be used. Indeed, such strategies have begun to be explored, using transmitter/founder virus envelopes^44^ and those derived from bNAb lineage studies^45^. We further suggest that immunization regimens with heterologous, cleaved Env trimers, given repeatedly over a prolonged period with adjuvants that improve innate stimulation, may enhance SHM and neutralization.

As long CDR H3 regions are a defining feature of bNAbs targeting the V1V2 epitope^5^,^8^, we explored how they were influenced by vaccination and infection. The adjuvants studied here had no influence on CDR H3 length. By contrast, and consistent with certain chronically infected HIV patients^25^, one of the SHIV_AD8_-infected animals (DCF1, a good neutralizer) developed antibodies with long CDR H3 regions that are anionic and predicted to have tyrosine sulfation as do human V1V2 bNAbs^25^,^26^. Although we cannot address the neutralization capacity of these antibodies without matched light chains, we explored the mechanism underlying the generation of CDR H3 length to understand how the NHP model compares to what is observed in humans. The finding that V(DD)J recombination contributed to such antibodies in DCF1 was notable as this mechanism is thought to be relatively rare^24^,^46^. The data presented here best support the generation of long CDR H3 regions by *N-*addition during V(D)J recombination, with only small insertions added rarely through SHM. Importantly, naive B cells with long CDR H3 regions were readily detectable in NHP as has been reported in the human naive repertoire^47^. Thus, in designing vaccines to specifically induce V1V2 antibodies, it should be possible to track the lineages of such long CDR H3 antibodies using the NHP model.

Finally, on the basis of alterations of the human B-cell repertoire after HIV and influenza infection^48^,^49^, we assessed the effects of Env vaccination and SHIV_AD8_ infection on V_H_ gene usage. Sundling *et al.*^50^ reported relative similarities in V_H_ gene usage between Env-specific and total memory B cells after vaccination, a finding confirmed here. However, studies have demonstrated that certain epitope specificities are preferentially derived from certain V_H_ genes (for example, VRC01-like class antibodies from V_H_1–2 (refs 5, 7, 9, 19)). In addition, HIV infection has been associated with increased V_H_1 family usage, most notably V_H_1–69 (refs 51, 52), whose NHP orthologue (*IGHV1E*) was found prominently in sequences from SHIV_AD8_-infected animals. In this study, the V_H_ genes *IGHV4A*, *4D* and *3J* were specifically associated with neutralizing responses following SHIV_AD8_ infection. Analysis of the V_H_ repertoire after Env protein/adjuvant immunization in two independent studies revealed that highly mutated sequences preferentially mapped to the *IGHV3Q* gene (whose human homologue gives rise to the CAP256-VRC26 family of bNAbs^25^). Furthermore, we note that the adjuvants induced differences in specific V_H_ genes, including *IGHV3Q*. Understanding the mechanisms by which adjuvants influence this process may be critical for strategies to rationally induce bNAbs.

In conclusion, on the basis of the similarities in innate and adaptive immune responses between macaques and humans, combined with the new draft Ig reference database and NGS platform, the NHP system allows for the evaluation of Env-specific B-cell development using future generations of HIV immunogens and adjuvant formulations.

## Methods

### SHIV_AD8_ infection and quantification of viral nucleic acids

Eight rhesus macaques of Indian origin ranging 2–8 years of age, either male or female, were maintained in accordance with the Association for Assessment and Accreditation of Laboratory Animal Care International standards, and were housed in a biosafety level 2 National Institute of Allergy and Infectious Diseases (NIAID) facility. Phlebotomies and sample collection were performed as previously described^53^. All animals were negative for the major histocompatibility complex class I Mamu-A*01 allele. Levels of CD4^+^ T-cell subsets were measured using flow cytometry as previously reported^22^. The origin and preparation of the tissue-culture-derived SHIV_AD8_ swarm and molecular cloned stocks have been previously described^21^,^54^. Viral RNA levels in the plasma were determined by real-time reverse transcription–PCR (ABI Prism 7900HT sequence detection system; Applied Biosystems) as previously reported^53^.

### Env vaccination study animals and immunizations

Fifty-three rhesus macaques of Indian origin ranging 3–8 years of age were divided into eight study groups of six and one group of five (Fig. 3a). This number of animals was chosen to give ∼90% power to detect a 1.0 log difference in antibody titres between two vaccine groups and 65% power to detect 0.5 log differences, given reasonable estimates of group s.d.'s from previous studies. Animals from previous vaccine studies were employed; these had not been exposed to any HIV or other retroviral proteins or nucleic acids and thus were naive for HIV Envelope; these were distributed evenly among the vaccine groups. For vaccination, 100 μg trimeric gp140 TV1ΔV2 protein (Env)^55^ was adsorbed to alum and administered as such, or co-adsorbed with an agonist of TLR 4 (an MPL derivative, E6020) or an agonist of TLR 7 (a proprietary benzonaphthyridine of Novartis, Cambridge, MA). Env was also mixed with MF59 or with a similar ANE formulated with the TLR 4 or 7 agonists (Novartis^56^). Finally, Env was mixed with pIC:LC (Hiltonol; Oncovir, Washington, DC) or Abisco100 ISCOMs (Isconova AB, Stockholm, Sweden). Animals were immunized intramuscularly in the quadriceps in a homologous prime-boost manner with Env alone, or with the adjuvant formulations at 0, 4, 12 and 24 weeks. Animals were housed and cared for at the animal facility of BIOQUAL Inc. (Rockville, MD) in accordance with the American Association for Accreditation of Laboratory Animal Care standards in accredited facilities, and all animal procedures were performed according to protocols approved by the Institutional Animal Care and Use Committees of the National Institute of Allergy and Infectious Diseases, National Institutes of Health.

### PBMC and plasma separation

Animals were bled into vacutainer tubes containing the anticoagulant and preservative, acid–citrate–dextrose. Blood was processed by Ficoll density centrifugation using Leucosep tubes (Greiner Bio-One, Monroe, NC). Plasma was then collected by aspiration above the resulting PBMC layer. PBMCs were transferred and washed with PBS before being cryopreserved in FBS+DMSO. Serum was collected by bleeding animals into serum-separating tubes that were then centrifuged.

### Titre ELISA

Immulon 4HBX plates (Thermo Scientific) were coated overnight at 4 °C with gp140 TV1ΔV2 protein diluted in carbonate buffer. Plates were then blocked with PBS+FBS, and plasma was applied in serial 10-fold dilutions and incubated at 37 °C. Detection was performed at room temperature with anti-monkey IgG-horseradish peroxidase (HRP; 1:30,000 Bethyl Labs) followed by TMB substrate-chromogen (Dako) and a 2N sulfuric acid stop solution. Washing was performed between steps with PBS+0.05% Tween 20 on an ELx405 automated plate washer (Bio-Tek Instruments). Plates were read on a SpectraMax Plus spectrophotometer (Molecular Devices), and data were analysed with the SoftMax Pro software (Molecular Devices).

### HIV-1 neutralization assay

Plasma samples were heat-inactivated at 56 °C for 1 h and centrifuged at 16,000*g* for 10 min to pellet precipitated lipoproteins. Neutralizing activity of plasma supernatants was assessed against Env-pseudotyped isolates using the TZM-bl luciferase reporter gene assay as described^57^,^58^,^59^. Plasma supernatants were diluted in a fourfold, 8-point dilution series, with a starting dilution of 1:20 after the supernatant was mixed with virus. Neutralization curves were fit using a five-parameter equation as described^59^. The amount of plasma required to inhibit infection by 50% is reported as the reciprocal 50% inhibitory dilution (ID_50_).

### Envelope protein probes

For bulk-sorting and NGS-sequencing of Env-specific B cells from SHIV_AD8_-infected NHP, CK15 3-3 gp120 Env protein^21^ was used to make a B-cell probe. For Env-specific B cells from Env-vaccinated NHP, TV1ΔV2 gp140 Env protein^55^ Env protein was used. These proteins were biotinylated at free primary amines using sulfo-NHS-LC-biotin (Pierce). The biotinylated Env was then complexed with ExtrAvidin-phycoerythrine (PE) or ExrAvidin-FITC (Sigma) in a 1:3 molar ratio of Env:ExtrAvidin. The ExtrAvidin was divided into five equal volumes and mixed sequentially for 20 min at 4 °C with the total Env portion. For single-cell sorting and Sanger sequencing of B cells, an Avi-tagged YU2 gp140 protein^60^ was expressed, purified and biotinylated using the biotin ligase Bir A (Avidity, Denver, CO). Biotinylation of the YU2 gp140 protein was confirmed using enzyme-linked immunosorbent assay (ELISA).

### B-cell culture

Memory B-cell culture protocol was adapted from previous reports^61^,^62^. Briefly, 1 day before culture, NIH 3T3 feeder cells expressing mouse CD40 ligand were seeded into 384-well plates at ∼6,500 cells per well with 100 U ml^−1^ IL-2 and 50 ng ml^−1^ IL-21 in IMDM with glutamax, 10% FBS and MycoZap (Lonza). The following day, PBMCs were stained with a fluorochrome-conjugated gp140 probe and positive events in the IgG^+^ gate were automatically sorted at one cell per well into the 384-well plates containing feeder cells. Plates were incubated for 2 weeks at 37 °C, 5% CO_2_. Supernatants were then applied at a 1:10 dilution to titre ELISA plates coated with anti-IgG (Rockland) or TV1 gp140. Bound IgG was detected with anti-IgG-HRP (Bethyl Laboratories) and read on a spectrophotometer.

### Flow cytometry phenotyping

PBMCs were stained for viability in PBS with Aqua Dead Cell Stain (Invitrogen) followed by staining for surface markers. For B-cell enumeration from PBMCs: IgD FITC (1:33, Southern Biotech, cat. no. 2030), CD20 Ax700 PE (1:100, clone 2H7), IgM PE Cy5 (1:50, BD Pharmingen, clone G20-127), gp140-biotin/ExtrAvidin PE (Sigma), CD3 APC Cy7 (1:100, BD Pharm., clone SP34-2), IgG APC (1:20, BD Pharm., clone G18-145), CD14 QD605 (1:300, clone M5E2), CD8 Pacific Blue (1:400, BD Pharm., clone RPA-T8); for B-cell sorting from vaccinated NHP: CD20 Ax700 PE (1:100, clone 2H7), IgM PE Cy5 (1:50, BD Pharm., clone G20-127), SA QD705, gp140-biotin/ExtrAvidin PE (Sigma), CD3 APC Cy7 (1:100, BD Pharm., clone SP34-2), IgG APC (1:20, BD Pharm., clone G18-145), CD14 QD605 (1:300, clone M5E2), CD8 Pacific Blue (1:400, BD Pharm., clone RPA-T8); for B-cell sorting from SHIV-infected NHP: CD20 Ax700 PE (1:100, clone 2H7), IgM PE Cy5 (1:50, BD Pharm.), gp120-biotin/ExtrAvidin FITC (Sigma), gp120-biotin/ExtrAvidin PE (Sigma), CD3 APC Cy7 (1:100, BD Pharm., clone SP34-2), IgG APC (1:20, BD Pharm., clone G18-145), CD14 QD800 (1:200, Life Technologies, clone TÜK4) and CD8 Pacific Blue (1:400, BD Pharm., clone RPA-T8).

Events were acquired on a BD LSR II flow cytometer (BD Biosciences) and FACS data were analysed using the FlowJo software (Tree Star). Gating trees for all populations studied are described in Supplementary Fig. 2.

### B-cell PCR

Antigen-specific B cells were sorted into OL-1 lysis buffer (Qiagen) and mRNA was extracted using an Oligotex Direct mRNA mini kit (Qiagen). RNA was then concentrated in a centrifugal concentrator (Millipore) and reverse transcription was performed using oligo-dT priming and Superscript II polymerase (Invitrogen). Multiplex 5′ PCR was performed to amplify the IgG H_C_; reactions proceeded for 35 cycles using an annealing temperature of 48 °C. Primers were derived from ref. 7, and include a five- to nine-nt barcode (all barcodes have a minimum three nt difference), and ‘XLR' sequences for 454 sequencing (Supplementary Table 5). PCR products were resolved on a 1.5% agarose gel, and the appropriate bands were excised and extracted. Antigen-specific samples were combined so that each sorted B cell would be sequenced at least 10-fold, given estimates of sequencer read outputs.

### RACE PCR technique

The majority of our data were generated using a standard multiplex primer PCR method employing 21 5′ primers (Supplementary Table 5), which have a variety of T_M_'s, thus potentially skewing our data set by preferential amplification with certain primers. To evaluate this, we compared our multiplex approach with the RACE technique, which uses a common 5′ primer to avoid primer bias for certain V_H_ genes. 5′ RACE ready cDNA was prepared using a SMARTer RACE cDNA Amplification Kit (Clontech) according to the manufacturer's directions and as previously described^63^; however, the 5′ CDS primer was added 30 min into the reverse transcription reaction. PCR was performed in two reactions (Supplementary Table 5); the latter used to add the XLR sequences. PCR products were resolved on a 1.5% agarose gel, and the appropriate bands were excised, extracted and sequenced by 454 pyrosequencing.

### New NHP Ig reference database

Genomic DNA was extracted from skin punches from 10 Rhesus macaques (of Indian origin) using the Agilent SureSelect gDNA Extraction Kit. One to three micrograms of the gDNA was then sheared using the Covaris E-Series Sample Preparation system into 150- to 200-base-pair fragments. gDNA libraries were prepared according to the protocol provided in the SureSelect XT Target Enrichment System Kit for Illumina Multiplexed Sequencing manual. The prepped DNA libraries were then mixed with the SureSelect Capture Library, which consists of biotinylated RNA library ‘baits' that are complimentary to the target region on the prepped library. Streptavidin-coated magnetic beads were attached to the baits that had hybridized to the target region and were then magnetically separated from the unbound DNA fraction. The baits were then digested, leaving only single-stranded target DNA. A final PCR was performed to amplify the captured DNA content and to add indices to the target DNA. The samples were then pooled in preparation for 100 base-pair, paired-end multiplexed sequencing on the Illumina HiSeq platform.

The resulting data were analysed, in part, with direct inference from 454 Ig transcripts, and annotated to form the reference database. Briefly, candidate V genes were identified on the basis of identification and evaluation of these essential features of functional V genes: recombination signals, in-frame coding sequence with invariant cysteine codons, leader regions and splice sites. A gene was classified as functional if it contained all of these essential features. If a putative V gene had no stop codons in the coding region but was missing one or more of the essential features of a V gene, it was classified as an open reading frame (ORF). Genes that contained stop codons in the coding region were classified as nonfunctional. The functional genes from all monkeys were divided into families on the basis of sequence homology to human V genes. Phylogenetic analysis was used to estimate whether sequence differences should be attributed to allelism or distinct genetic loci. Because the organization of these genes on the locus could not be determined, numbers were not used in the gene names, since numbers are intended to indicate the gene's position on the chromosome. Instead, letters were used. Alleles are indicated as usual by an integer following an asterisk. The database used in the analysis contained all and only the genes that were annotated as functional or an ORF. GenBank accession codes for these V_H_ gene sequences are given in Supplementary Table 1.

To compare this reference database with the previous rhesus database and that for humans, two phylogenetic trees were reconstructed using PhyML (Supplementary Fig. 3a,b). The human-NHP gene dendrogram was reconstructed with TN93 substitution model; the NHP previous-new database dendrogram was built with the GTR substitution model. The optimal substitution models were estimated using MEGA5 (ref. 64); sequences were aligned using Muscle^65^. All available sequences and alleles were used for analysis.

### 454 deep sequencing

Sequencing was performed by the NIH Intramural Sequencing Center (NISC). For each sample, the amplicon library concentrations were determined with quantitative PCR (qPCR) using Library Quantification Kit −454 Titanium (Lib-L)/Universal (Kapa Biosystems) on a CFX96 Real-Time System (Bio-Rad). The libraries were then amplified on Capture Beads by emulsion PCR using a ratio of DNA molecules: DNA Capture Bead between 0.5 and 2.0 and GS Titanium emPCR Kit (Lib-L) and emPCR Breaking Kits (Roche) following the manufacturer's instructions. Approximately 0.7–1.0 × 10^6^ enriched Capture Beads were applied to each region of a two-region PicoTiterPlate (70 × 75) and sequenced on a GS FLX Sequencer (Roche-454 Life Sciences, Bradford, CT) using GS FLX Titanium Sequencing Kit for 200 cycles. Post-run signal processing, quality filtering (modified according to recommendations in Roche-454 Life Sciences Application Brief No. 001-2010) and conversion of flowgram intensities to nucleotide sequence and Phred-based quality scores were performed on an off-instrument linux cluster running 454 application software version 2.6.

### Bioinformatic analysis of sequencing data

A data set of high-quality unique H_C_ sequences was collected using the following pipeline. First, raw reads were sorted according to the barcodes used for different time points or different animals, with one nucleotide mismatch allowed in the barcode. Next, reads with length <300 or >700 bp were removed. Germline V gene and J gene were assigned for each read using BLASTN with E value cutoff 1E-10 and 1E-3, respectively^66^. 5′ primers and 3′ primers were removed on the basis of the assigned V and J gene boundary. Because single-nucleotide insertions and deletions are common in 454 pyrosequencing, we partially rectified high confidence single-nucleotide insertions and deletions in the V segment using the following method: each read and its assigned germline V gene were aligned using Muscle^65^; single-nucleotide insertions and deletions were identified as a single gap in the aligned germline gene and read sequence, respectively. If the 5′ two nucleotides and 3′ two nucleotides of an insertion position mapped exactly to the germline V gene, the insertion was removed; if the 5′ two nucleotides and 3′ two nucleotides of a deletion position were a perfect match between read and germline V gene, the aligned germline nucleotide at the deletion position was used to rectify this deletion. However, reads were excluded if they contained more than three single-nucleotide deletions and/or insertions. Next, the correct ORF of a read was identified as the one that showed the highest amino-acid sequence identity to the germline V gene (only the three forward ORFs were compared). Furthermore, if the ratio of amino-acid identity between the read and germline V gene divided by nucleotide identity was lower than 0.7, the read was marked as having a frameshift. High-quality reads were then selected using the following criteria: no more than a 15-nucleotide truncation at the 5′ terminus of the V region, no stop codon and no frameshift. Finally, a data set of unique H_C_ sequences was collected by removing PCR and sequencing redundancy among the high-quality reads using CD-HIT with a sequence identity cutoff of 0.975 and a length coverage cutoff of 0.99 (ref. 18).

To calculate divergence in the CDR and FR of each V segment, we first aligned amino-acid sequences of each antibody V segment and its assigned germline V gene using Muscle. The boundaries of the CDRs and FRs from germline genes were used to determine the corresponding boundaries of the sequence. The CDR and FR boundaries of germline V genes were assigned using the IMGT database^67^ and were manually inspected. The nucleotide-level divergence was then calculated. The amino-acid sequence alignment in each region was further used as a guide to align the sequence to its assigned germline gene at the DNA level so that divergence at the nucleotide level could be calculated. The CDR3 H3 sequence was extracted starting from the residue next to the second conserved cysteine of the V segment to the residue before the conserved H_C_ W-G-X-G motif in the J gene where X represents any amino acid.

Long CDR H3 (≥28 aa) antibodies found in animal DCF1 were grouped into 14 lineages using the following parameters: all antibodies within a lineage have the same V and J assignments, CDR H3 identity ≥80% and ≤40% variation in CDR H3 length. All lineages were manually checked to exclude PCR crossover and sequencing error. The phylogenetic tree (Fig. 2g) was built using PhyML with GTR substitution model, which is the best model estimated by MEGA5. Tyrosine sulfation was predicted using the GPS-TSP programme ( http://tsp.biocuckoo.org). ProteinCaculator was used for charge calculations at pH 7 ( http://protcalc.sourceforge.net).

### SHIV_AD8_ viral sequencing

SGA was performed on blood plasma from eight SHIV_AD8_-infected animals sampled at weeks 6, 26, 54 and 99 except where indicated. For viral RNA extraction and cDNA synthesis, from each plasma specimen at least 20,000 viral RNA copies were extracted using the QIAamp Viral RNA Mini kit (Qiagen). Reverse transcription of RNA to single-stranded cDNA was performed using SuperScript III reverse transcription according to the manufacturer's recommendations (Invitrogen). In brief, a cDNA reaction of 1 × RT buffer, 0.5 mM of each deoxynucleoside triphosphate, 5 mM dithiothreitol, 2 U ml^−1^ RNaseOUT (RNase inhibitor), 10 U ml^−1^ of SuperScript III reverse transcription and 0.25 mM antisense primer SIVEnvR1 5′-TGTAATAAATCCCTTCCAGTCCCCCC-3′ was incubated at 50 °C for 60 min, 55 °C for 60 min and then heat-inactivated at 70 °C for 15 min followed by treatment with 2 U of RNase H at 37 °C for 20 min. The newly synthesized cDNA was used immediately or frozen at −80 °C. For SGA of SHIV Env, a 3.3-kb fragment that includes the entire *env* gene was sequenced from each animal at various time points following infection using a limiting dilution PCR; therefore, only one amplifiable molecule is present in each reaction. SGA was performed by serially diluting cDNA distributed among independent PCR reactions to identify a dilution where amplification occurred in <30% of the total number of reactions. PCR amplification was performed with 1 × PCR buffer, 2 mM MgSO_4_, 0.2 mM of each deoxynucleoside triphosphate, 0.2 μM of each primer and 0.025 U μl^−1^ Platinum Taq High Fidelity polymerase (Invitrogen) in a 20-μl reaction. First-round PCR was performed with primer SIVEnvF1 5′-CCTCCCCCTCCAGGACTAGC-3′ and antisense primer SIVEnvR1 under the following conditions: one cycle of 94 °C for 2 min, 35 cycles at 94 °C for 15 s, 55 °C for 30 s and 68 °C for 5 min, followed by a final extension of 68 °C for 10 min. Next, 1 μl from the first-round PCR product was added to a second-round PCR reaction that included the sense primer SHIVEnvF2 5′-GACCTCCAGAAAATGAAGGACCAC-3′ and antisense primer SIVEnvR2 5′-ATGAGACATRTCTATTGCCAATTTGTA-3′ performed under the same conditions used for first-round PCR, but with a total of 45 cycles. Correct sized amplicons were identified using agarose gel electrophoresis and directly sequenced with second-round PCR primers and nine HIV-specific primers using Big Dye Terminator Technology (Applied Biosystems). To confirm PCR amplification from a single template, chromatograms were manually examined for multiple peaks, indicative of the presence of amplicons resulting from PCR-generated recombination events, *Taq* polymerase errors or multiple variant templates.

All 441 sequences were aligned using ClustalW and phylogenetic trees were constructed using the neighbour-joining method. Diversity measurements were determined at each time point for each animal using pair-wise comparisons. Similarly, divergence measurements were determined at each time point for each animal using the centre of the tree as the root. Viral sequences were aligned using Muscle with manual correction. Sequences logos for the V2 and V3 regions were generated by Weblogo^68^. All sequences were deposited in GenBank with accession codes KM082525 to KM082965.

To estimate the SHIV evolutionary rate within each host, we first confirmed that no recombination took place in the ENV gene using RPD3 (ref. 69). The HKY+gamma+I substitution model was used, having been evaluated by MEGA5 to fit the sequence substitution pattern. The evolutionary rate was then estimated using Beast with 10^8^ Markov chain simulations^70^. During simulation, a relaxed log-normal molecular clock was selected for evaluating longitudinal rate variation and Bayesian Skyline plot method was used to model virus population dynamics. The first 10^7^ runs were used as burn in and discarded before rate estimation.

### Statistical analysis and sample blinding

Data were graphed and analysed with the Prism software (GraphPad). For nonsequence data, a Kruskal–Wallis test with a Dunn's correction for multiple comparisons was used to compare means among vaccine groups, using the ‘Env+alum' group as a control. A two-way analysis of variance test with a Bonferroni correction for multiple comparisons was employed to analyse vaccine groups across multiple time points, using the ‘pre' time point as a control. For sequencing data, a mixed effect linear regression model, allowing for random effects for each animal, was employed to generate *P* values. Comparisons of V_H_ gene proportions and correlations of V_H_ genes to the neutralization status were performed using the *χ*^2^-test with Yates' correction or Fisher's exact test. SHIV viral sequencing data were compared with multiple *t*-tests using a false discovery rate (*Q*)=1%. Investigators assembling data collected were not blinded. Plasma and PBMC samples were analysed for viral loads, viral sequences, antibody responses and B-cell sequences in four geographically separate laboratories. Each laboratory was blinded with regard to the results from the other laboratories.

## Author contributions

J.R.F., Z.S., L.S. and R.A.S. conceived the study and wrote the paper. N.M.V., P.M., E.D.G., S.W.B., M.S. and D.T.O. developed the vaccine formulations. J.R.F., Y.N., S.D.S., B.J.F., T.Y., R.M.-N. and R.M.L. designed and carried out experiments. B.F.K. performed the viral sequencing. NISC performed the 454 sequencing. Z.S., Z.Z. and L.S. performed the 454 bioinformatic analyses. S.D., D.W., B.F.H. and D.D. resequenced the macaque Ig locus. A.R. and T.B.K. analysed the macaque DNA sequences. R.A.K., J.R.M., M.A.M. and R.A.S. gave conceptual advice and project oversight.

## Additional information

**Accession codes**: The SHIV viral sequences have been deposited in the GenBank database with accession codes KM082525 to KM082965. Novel NHP Ig heavy chain sequences have been deposited in the GenBank database with accession codes KP710506 to KP710583.

**How to cite this article:** Francica, J.R. *et al.* Analysis of immunoglobulin transcripts and hypermutation following SHIV_AD8_ infection and protein-plus-adjuvant immunization. *Nat. Commun.* 6:6565 doi: 10.1038/ncomms7565 (2015).

## Supplementary Material

Supplementary Information Supplementary Figures 1-9 and Supplementary Tables 1-5
