# Supplementary material for: Analysis of immunoglobulin transcripts and hypermutation following SHIVAD8 infection and protein-plus-adjuvant immunization
Source: Nat Commun. 2015 Apr 10;6:6565. doi: 10.1038/ncomms7565 (PMC4403371; doi:10.1038/ncomms7565)
Supplement: Supplementary Information — Supplementary Figures 1-9 and Supplementary Tables 1-5 [file ncomms7565-s1.pdf]

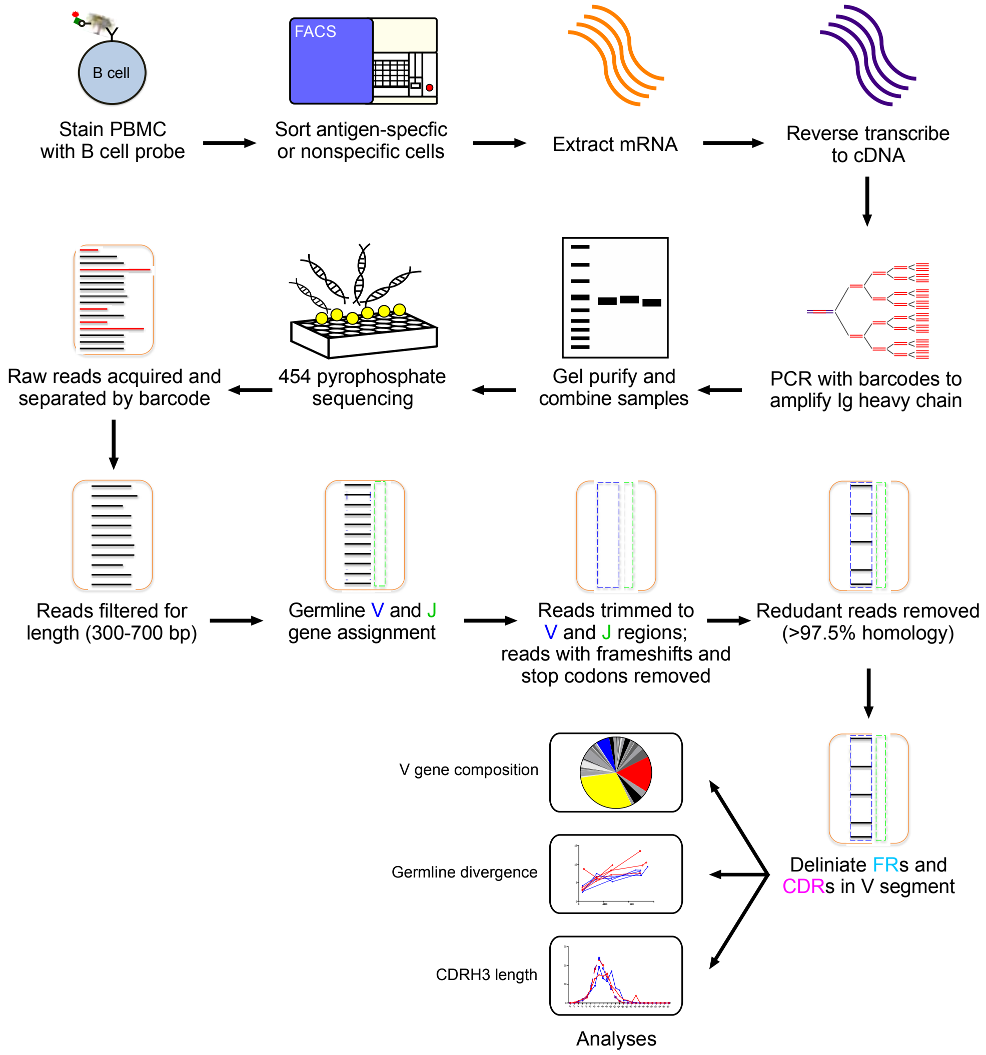

Supplementary Figure 1. Experimental pipeline for B cell deep sequencing and bioinformatic analysis. Antigen specific B cells were sorted based on binding to a gp140 or gp120 probe. mRNA was extracted and reverse transcribed to cDNA for Ig H_C amplification. Unique barcodes are incorporated at the 3' end of amplicons to differentiate between animals and time points. Products were then gel purified and combined for 454 pyrophosphate sequencing. Raw reads were separated by barcode, filtered for size, then given a V and J assignment. These reads were then trimmed of the primers, and insertions and deletions were corrected where possible; reads with uncorrectable frameshifts and stop codons were removed. Finally, redundant reads were removed and FR and CDR regions were delineated; subsequent analyses of SHM, V_H gene composition and CDR H3 length were then performed.

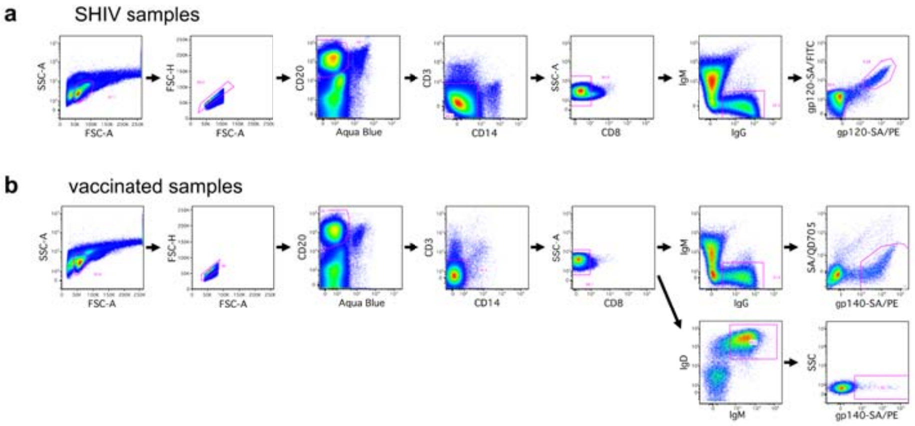

Supplementary Figure 2. Flow cytometry gating strategies. (**a**) Antigen specific and nonspecific B cell gating from SHIV infected NHP PBMCs. Antigen specific memory cells are defined as IgM-/IgG+/gp140 FITC+/gp140 PE+. Non-specific memory cells are defined as IgM-/IgG+/gp140 FITC-/gp140 PE-. (**b**) Ag-specific B cell gating from Env vaccinated NHP PBMCs. Antigen-specific memory cells are defined as IgM-/IgG+/SA-/gp140+. Antigen-specific naïve cells are defined as IgM+/IgD+/gp140 PE+. To confirm our ability to isolate Env-specific B cells, probe positive memory B cells were single-cell sorted from PBMCs collected after Env vaccination. These were differentiated into antibody-secreting cells and supernatants were tested for reactivity to Env. Of these gp140+ memory B cells, 340/605 (56%) produced detectible IgG, of which 318 (94%) reacted with Env. A small number of probe positive events were detected in pre-vaccination samples, but only 2/41 (5%) of such cells reacted with Env. These data confirm that the vast majority of the Env probe-sorted cells are Env-specific.

a

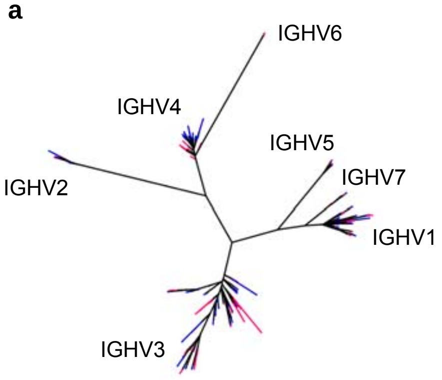

Human verses new NHP
reference databases

b

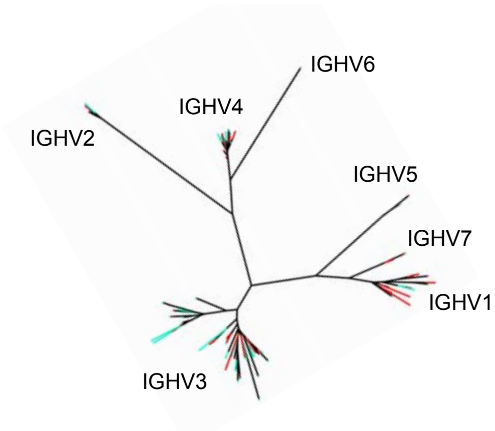

New NHP verses previous NHP
reference databases

c

New NHP V_H gene	Human V_H gene	Previous NHP V_H gene	Human vs. New NHP homolog identity (nt)	Human vs. New NHP homolog identity (aa)	Previous NHP vs. New NHP homolog identity (nt)	Previous NHP vs. New NHP homolog identity (aa)
IGHV1-A	IGHV1-18	VH1.23	90.62	81.25	90.62	85.42
IGHV1-C	IGHV1-46	VH1.16	89.58	84.38	100	100
IGHV1-D	IGHV1-46	VH1.36	90.28	84.38	89.24	82.29
IGHV1-E	IGHV1-69	VH1.36	94.79	90.62	100	100
IGHV1-F	IGHV1-8	VH1.61	92.01	90.62	93.75	85.42
IGHV1-G	IGHV1-2	VH1.23	92.36	89.58	100	100
IGHV1-H	IGHV1-45	VH1.23	92.68	87.23	90.24	80.85
IGHV1-I	IGHV1-24	VH1.59	95.47	91.67	100	100
IGHV1-J	IGHV1-F	VH1.53	84.72	89.58	100	100
IGHV2-A	IGHV2-5	VH2.12	96.22	93.81	100	100
IGHV2-B	IGHV2-70	VH2.25	95.53	92.78	100	100
IGHV2-C	IGHV2-70	VH2.3	96.22	97.94	100	100
IGHV3-A	IGHV3-11	VH3.15	90.24	86.46	100	100
IGHV3-B	IGHV3-74	VH3.2	89.93	85.42	100	100
IGHV3-C	IGHV3-11	VH3.15	90.24	85.42	95.83	92.71
IGHV3-D	IGHV3-NL1	VH3.8	90.59	85.42	100	100
IGHV3-E	IGHV3/OR16-10	VH3.170	83.04	72.63	100	100
IGHV3-F	IGHV3-74	VH3.15	94.79	93.75	91.32	88.54
IGHV3-G	IGHV3-66	VH3.55	87.37	76.84	100	100
IGHV3-H	IGHV3-48	VH3.50	91.32	89.47	100	100
IGHV3-I	IGHV3-23	VH3.58	93.06	95.79	95.49	94.74
IGHV3-J	IGHV3-23	VH3.63	93.4	94.79	99.65	98.96
IGHV3-K	IGHV3-23	VH3.58	91.67	90.43	100	100
IGHV3-L	IGHV3-48	VH3.33	91.67	90.62	100	100
IGHV3-M	IGHV3-9	VH3.17	93.75	90.62	88.89	86.46
IGHV3-N	IGHV3/OR16-10	VH3.27	93.68	92.39	89.05	85.87
IGHV3-AB	IGHV3-48	VH3.17	90.62	86.46	100	100
IGHV3-O	IGHV3-48	VH3.13	89.24	85.11	100	100
IGHV3-P	IGHV3-48	VH3.4	92.36	90.43	100	100
IGHV3-Q	IGHV3-33	VH3.44	93.38	90.62	100	100
IGHV3-R	IGHV3-11	VH3.14	87.46	80.21	100	100
IGHV3-S	IGHV3/OR15-7	VH3.5	94.22	90.82	100	100
IGHV3-T	IGHV3-72	VH3.47	95.92	94.9	100	100
IGHV3-U	IGHV3-73	VH3.29	95.24	93.88	100	100
IGHV3-V	IGHV3-15	VH3.9	92.49	90.82	100	100
IGHV3-W	IGHV3-15	VH3.6	91.84	86.73	100	100
IGHV3-X	IGHV3-49	VH3.10	92.18	91.84	100	100
IGHV3-Y	IGHV3-49	VH3.30	92.52	89.8	100	100
IGHV3-Z	IGHV3-49	VH3.42	88.44	83.67	98.3	96.94
IGHV3-AA	IGHV3-49	VH3.18	92.86	92.86	100	100
IGHV4-A	IGHV4/OR15-8	VH4.57	93.81	91.67	99.66	100
IGHV4-B	IGHV4-59	VH4.22	93.4	91.58	94.44	89.58
IGHV4-C	IGHV4-59	VH4.38	91.75	89.47	94.85	87.63
IGHV4-D	IGHV4-B	VH4.38	80.07	90.62	100	100
IGHV4-E	IGHV4-61	VH4.28	94.5	93.81	100	100
IGHV4-F	IGHV4-B	VH4.34	80.41	94.79	100	100
IGHV4-G	IGHV4/OR15-8	VH4.35	92.78	88.54	100	100
IGHV4-H	IGHV4/OR15-8	VH4.40	92.44	90.62	100	100
IGHV4-I	IGHV4-B	VH4.40	78.35	90.62	94.5	91.75
IGHV4-J	IGHV4-61	VH4.39	92.86	91.67	95.19	91.75
IGHV4-K	IGHV4-59	VH4.37	94.44	94.74	97.57	93.75
IGHV4-L	IGHV4-4	VH4.11	93.4	89.47	100	100
IGHV5-A	IGHV5-51	VH5.7	95.83	92.71	100	100
IGHV5-B	IGHV5-51	VH5.46	94.79	88.54	99.65	98.96
IGHV5-C	IGHV5-51	VH5.20	93.06	86.32	100	100
IGHV6-A	IGHV6-1	VH6.1	95.29	92.93	100	100
IGHV7-A	IGHV7-81	VH7.21	94.44	90.62	100	100
IGHV7-B	IGHV7-4-1	VH7.21	93.4	87.5	92.01	87.5

Supplementary Figure 3. New NHP draft reference genome. Dendrograms are shown comparing the new NHP IGVH reference database to (**a**) the human database and (**b**) the previous NHP database. (**c**) Individual NHP V_H genes are shown with their nearest human ortholog, including the sequence similarity at the nucleotide or amino acid level.

a

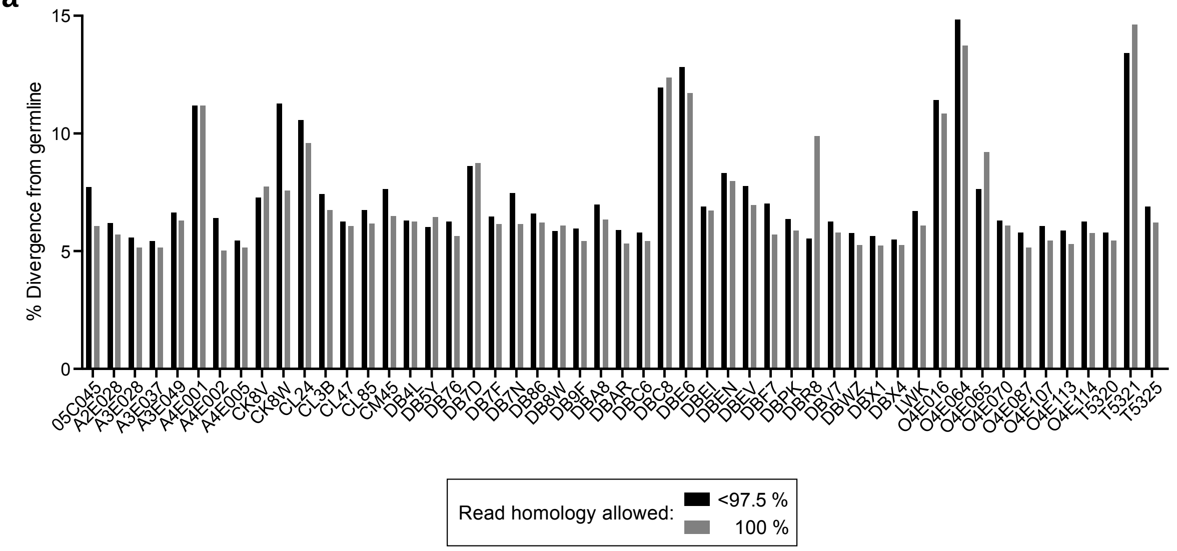

b

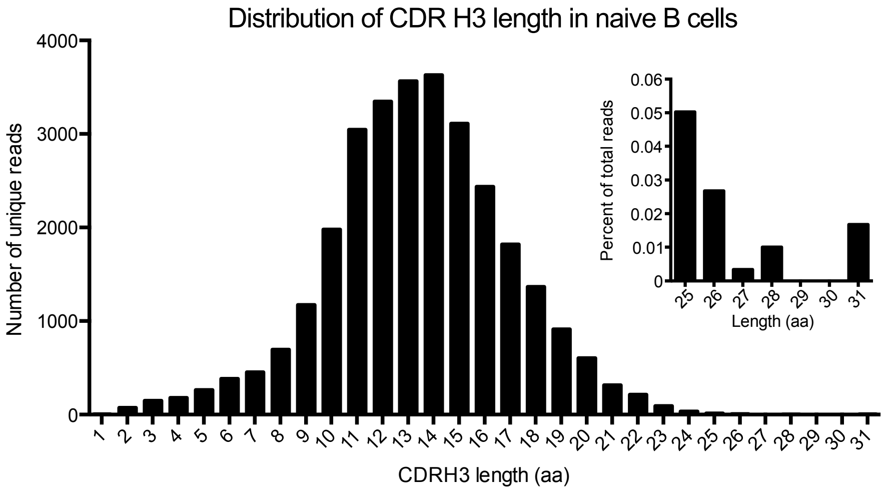

Supplementary Figure 4. 454 sequencing of Env vaccinated NHP. (**a**) Effect of sequence homology cutoff on mean SHM. Highly-homologous reads (above 97.5%) from vaccinated animals were removed (black bars) or included (grey bars) and used to calculate mean SHM. (**b**) Distribution of CDR H3 lengths in naïve B cells. IgM sequences were amplified by RACE from ~6.7e5 sorted naïve B cells from 2 NHPs before vaccination; 29,874 unique sequences were obtained. Graphs show the frequency distribution of CDR H3 lengths by number of sequences, or by percentage of the total dataset, for reads ≥25 aa.

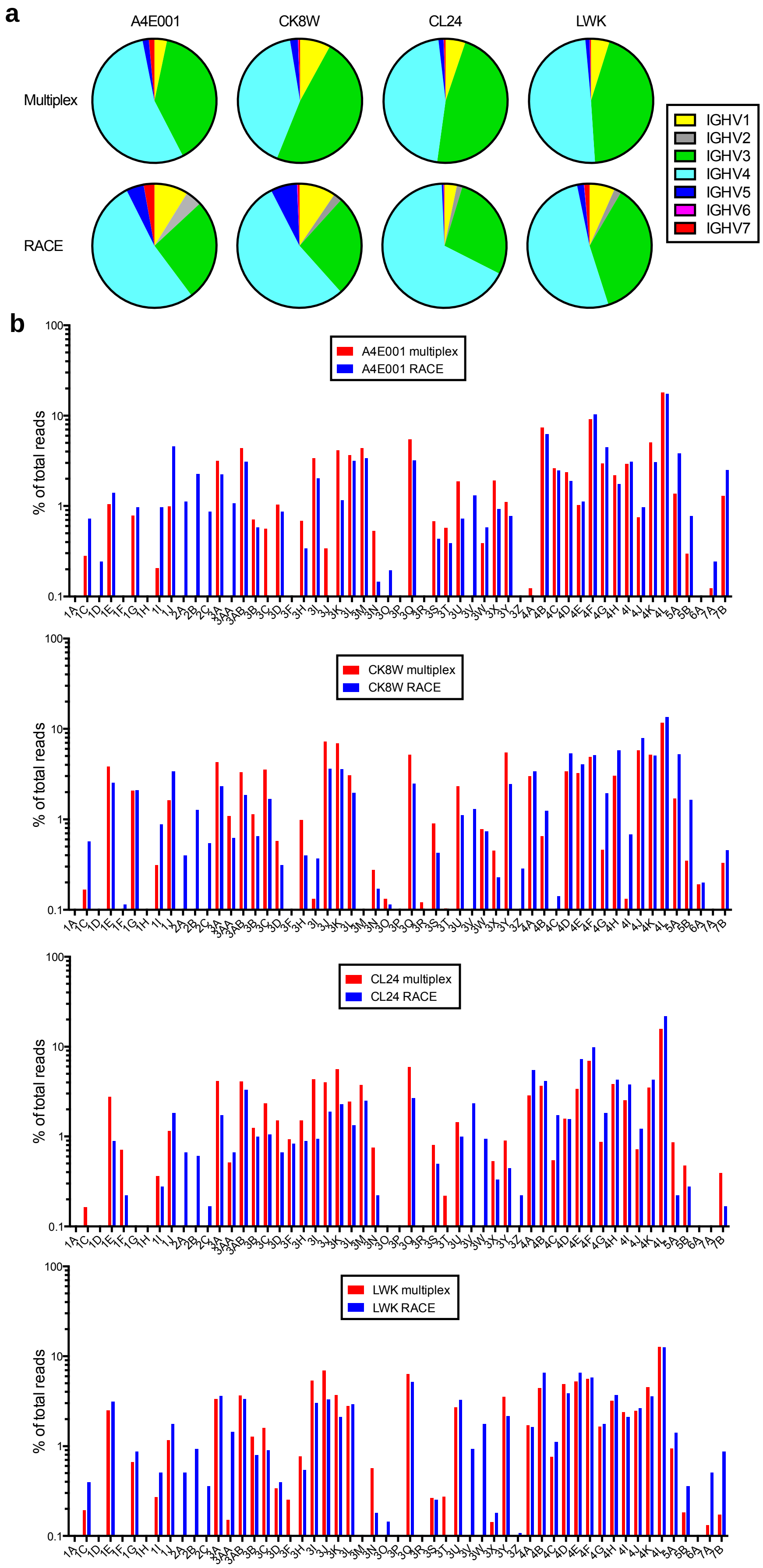

a
A4E001
CK8W
CL24
LWK
Multiplex
RACE
IGHV1
IGHV2
IGHV3
IGHV4
IGHV5
IGHV6
IGHV7
b
A4E001 multiplex
A4E001 RACE
CK8W multiplex
CK8W RACE
CL24 multiplex
CL24 RACE
LWK multiplex
LWK RACE
% of total reads
100
10
1
0.1

Supplementary Figure 5. Multiplex and RACE PCR approaches show similar V_H gene compositions. Bulk PBMC samples from 4 animals before vaccination were amplified using the standard 5' multiplex primer approach, or the RACE method, and analyzed by deep sequencing. (**a**) The frequency of each V_H family is represented as a fraction of the total. Note the presence IGHV2 sequences, which were omitted from the multiplex primer set. (**b**) The percent composition of each V_H gene is depicted for the multiplex and RACE methods to observe potential biased amplification of specific V_H genes. Each graph shows data from a different animal. Note that a small number of V_H2 sequences were amplified, along with sequences that mapped to IGHV 3V and 3Z, which were not detected in the multiplex datasets.

a

ID50	Clade/ type	A						AG	B																	C							Other					
	Tier	2	1B	2	2	2	2	1B	1B	1	2	2	2	2	2	2	2	2	2	2	2	2	1	1B	1B	2	1A	2	2	2	2	1B						
Animal ID	Weeks post infection	KER2018.11	Q23.17	Q168.a2	Q769.h5	RW020.2	UG037.8	DJ263.8	6535.3	BaL.01	6101.10	BG1168.01	CAAN.A2	JR-FL	JR-CSF.JB	PVO.4	THRO4156.18	TRJO4551.58	TRO.11	YU2	7165.18	AC10.0.29	ADA.DG	HXB2	SS1196.1	CAP45.2.00.G3	MW965.26	DU156.12	DU422.01	ZA012.29	ZM55.28a	ZM106.9	SHIVDH12 clone 7 V3 AD8	SHIVAD8 CK15 3-3	# HIV-1 neutralized	# HIV-1 tested	% breadth	Geometric mean
**Poor responders**																																						
DBJI	6	<20	<20	<20	<20	<20	<20	<20	<20	<20	<20	<20	<20	<20	<20	<20	<20	<20	<20	<20	<20	<20	<20	<20	<20	<20	<20	<20	<20	<20	<20	<20	48	<20	0	31	0	n/a
	26	<20	<20	<20	<20	<20	<20	<20	<20	32	<20	<20	<20	<20	<20	<20	<20	<20	<20	<20	<20	<20	<20	72	32	<20	502	<20	<20	<20	<20	<20	448	<20	2	31	6	190
	54	<20	<20	<20	<20	<20	<20	<20	<20	21	<20	<20	<20	<20	<20	<20	<20	<20	<20	<20	<20	<20	96	79	120	<20	376	<20	<20	<20	<20	<20	616	<20	4	31	13	136
	100	<20	<20	<20	<20	<20	<20	<20	<20	32	<20	<20	<20	<20	<20	<20	<20	<20	<20	<20	<20	<20	47	104	96	<20	654	<20	<20	<20	<20	<20	414	<20	4	31	13	132
DCV9	6	<20	<20	<20	<20	<20	<20	<20	<20	<20	<20	<20	<20	<20	<20	<20	<20	<20	<20	<20	<20	<20	<20	<20	<20	<20	<20	<20	<20	<20	<20	<20	<20	<20	0	31	0	n/a
	28	<20	<20	<20	<20	<20	<20	<20	<20	<20	<20	<20	<20	<20	<20	<20	<20	<20	<20	<20	<20	<20	<20	<20	<20	<20	<20	<20	<20	<20	<20	<20	116	<20	0	31	0	n/a
	55	<20	<20	<20	<20	<20	<20	<20	<20	<20	<20	<20	<20	<20	<20	<20	<20	<20	<20	<20	<20	<20	<20	80	34	<20	95	<20	<20	<20	<20	<20	774	<20	2	31	6	87
	102	<20	<20	<20	<20	<20	<20	<20	<20	29	<20	<20	<20	<20	<20	<20	<20	<20	<20	<20	<20	<20	<20	786	108	<20	229	<20	<20	<20	<20	<20	1,234	<20	3	31	10	269
DBVC	6	<20	<20	<20	<20	<20	<20	<20	<20	<20	<20	<20	<20	<20	<20	<20	<20	<20	<20	<20	<20	<20	<20	<20	<20	<20	<20	<20	<20	<20	<20	<20	<20	<20	0	31	0	n/a
	26	<20	<20	<20	<20	<20	<20	<20	<20	<20	<20	<20	<20	<20	<20	<20	<20	<20	<20	<20	<20	<20	<20	<20	<20	<20	26	<20	<20	<20	<20	<20	43	<20	0	31	0	n/a
	54	<20	<20	<20	<20	<20	<20	<20	<20	<20	<20	<20	<20	<20	<20	<20	<20	<20	<20	<20	<20	<20	<20	<20	22	<20	81	<20	<20	<20	<20	<20	145	<20	1	31	3	81
	100	<20	<20	<20	<20	<20	<20	<20	<20	20	<20	<20	<20	<20	<20	<20	<20	<20	<20	<20	<20	<20	<20	<20	33	<20	156	<20	<20	<20	<20	<20	194	<20	1	31	3	156
DBZ3	6	<20	<20	<20	<20	<20	<20	<20	<20	<20	<20	<20	<20	<20	<20	<20	<20	<20	<20	<20	<20	<20	<20	<20	<20	<20	<20	<20	<20	<20	<20	<20	<20	<20	0	31	0	n/a
	26	<20	<20	<20	<20	<20	<20	<20	<20	<20	<20	<20	<20	<20	<20	<20	<20	<20	<20	<20	<20	<20	<20	<20	<20	<20	<20	<20	<20	<20	<20	<20	27	<20	0	31	0	n/a
	54	<20	<20	<20	<20	<20	<20	<20	<20	<20	<20	<20	<20	<20	<20	<20	<20	<20	<20	<20	<20	<20	<20	<20	<20	<20	25	<20	<20	<20	<20	<20	146	<20	0	31	0	n/a
	100	<20	<20	<20	<20	<20	<20	<20	<20	67	<20	<20	<20	<20	<20	<20	<20	<20	<20	<20	<20	<20	<20	609	211	<20	454	<20	<20	<20	<20	<20	975	<20	4	31	13	250
**Good responders**																																						
DC6W	6	<20	<20	<20	<20	<20	<20	<20	<20	<20	<20	<20	<20	<20	<20	<20	<20	<20	<20	<20	<20	<20	<20	<20	<20	<20	<20	<20	<20	<20	<20	<20	58	<20	0	31	0	n/a
	28	<20	<20	<20	<20	<20	<20	24	47	313	<20	<20	<20	<20	112	<20	<20	<20	<20	<20	<20	<20	<20	464	247	<20	1,466	<20	<20	<20	<20	<20	1,548	<20	6	31	19	255
	55	<20	<20	<20	<20	<20	<20	298	153	1,806	<20	<20	<20	<20	2,512	<20	<20	<20	<20	<20	<20	<20	3,294	4,301	1,321	<20	12,405	24	<20	<20	<20	<20	6,330	67	8	31	26	1,622
	102	<20	<20	<20	<20	<20	<20	825	382	1,486	<20	<20	<20	29	2,247	<20	<20	<20	53	21	29	<20	4,206	7,375	2,554	<20	16,005	85	<20	<20	<20	<20	5,086	148	10	31	32	1,196
DC8T	6	<20	<20	<20	<20	<20	<20	<20	<20	<20	<20	<20	<20	<20	<20	<20	<20	<20	<20	<20	<20	<20	<20	<20	<20	<20	<20	<20	<20	<20	<20	<20	<20	<20	0	31	0	n/a
	28	<20	<20	<20	<20	<20	<20	<20	<20	126	<20	<20	<20	<20	24	<20	<20	<20	<20	<20	<20	<20	<20	172	99	<20	421	<20	<20	<20	<20	<20	1,042	<20	4	31	13	173
	55	<20	<20	<20	<20	<20	<20	<20	<20	94	<20	<20	<20	<20	41	<20	<20	<20	<20	<20	<20	<20	755	504	420	<20	4,872	<20	<20	<20	<20	<20	4,977	78	6	31	19	380
	102	<20	155	<20	<20	30	<20	277	60	200	<20	<20	<20	<20	163	<20	<20	<20	<20	94	<20	<20	2,123	782	643	<20	19,831	248	<20	<20	<20	<20	11,235	77	11	31	35	400
DCF1	8	<20	<20	<20	<20	<20	<20	<20	<20	<20	<20	<20	<20	<20	<20	<20	<20	<20	<20	<20	<20	<20	<20	<20	<20	<20	74	<20	<20	<20	<20	<20	75	<20	1	31	3	74
	28	<20	<20	<20	<20	<20	<20	<20	42	359	<20	<20	<20	21	386	<20	<20	<20	<20	<20	<20	<20	<20	233	321	<20	3,385	29	<20	<20	<20	<20	2,041	20	6	31	19	337
	55	<20	<20	<20	<20	<20	<20	60	60	586	<20	<20	<20	817	334	<20	<20	61	<20	<20	<20	<20	1,291	2,106	1,300	<20	5,287	95	<20	<20	<20	<20	3,990	81	11	31	35	415
	102	<20	<20	<20	<20	<20	<20	285	223	660	<20	<20	<20	606	416	<20	<20	<20	<20	<20	<20	<20	2,697	6,450	1,672	<20	7,671	<20	<20	<20	<20	<20	2,555	25	9	31	29	1,100
DCC7	6	<20	<20	<20	<20	<20	<20	<20	<20	<20	<20	<20	<20	<20	<20	<20	<20	<20	<20	<20	<20	<20	<20	<20	<20	<20	<20	<20	<20	<20	<20	<20	61	<20	0	31	0	n/a
	26	<20	<20	<20	<20	<20	<20	148	98	283	<20	<20	<20	<20	65	<20	<20	<20	<20	<20	<20	<20	<20	1,797	146	<20	6,590	<20	<20	<20	<20	<20	1,252	73	7	31	23	334
	54	<20	<20	<20	<20	<20	<20	213	394	127	<20	<20	<20	<20	<20	<20	<20	<20	<20	<20	<20	<20	371	1,417	200	<20	10,358	<20	<20	<20	<20	<20	622	87	7	31	23	529
	100	<20	<20	<20	<20	<20	<20	271	137	161	<20	<20	<20	94	<20	<20	<20	<20	<20	<20	<20	<20	747	5,633	175	<20	5,292	<20	<20	<20	<20	<20	862	59	8	31	26	465

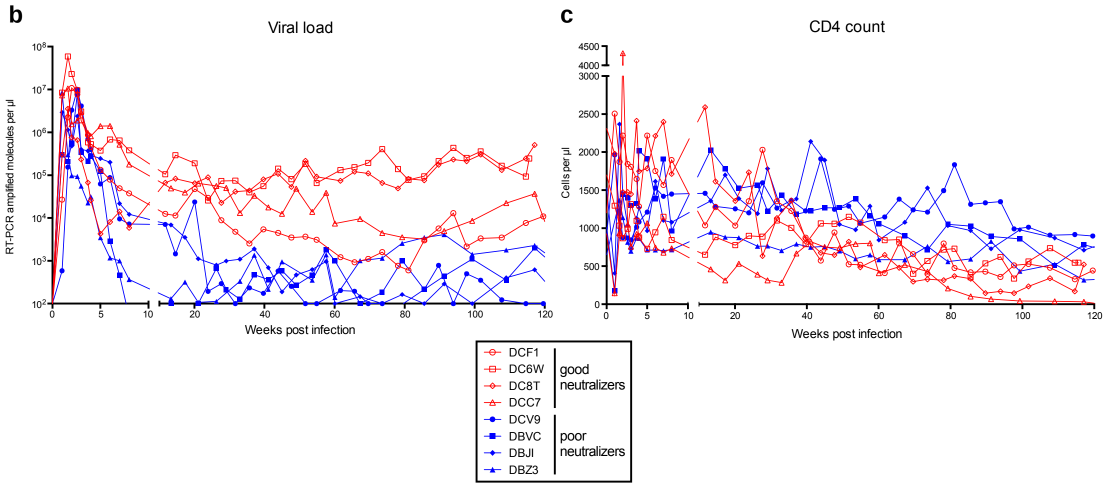

Supplementary Figure 6. Characteristics of $SHIV_{AD8}$-infected NHP. (**a**) Extended virus neutralization panel using plasma from multiple time points post $SHIV_{AD8}$ infection. ID_{50} values are shown; colors indicate potency: 40-99, green; 100-999, yellow; ≥1000, red. Percent breadth is calculated as (# HIV-1 neutralized / # HIV-1 tested)*100%; geometric means were calculated on values ≥40. (**b**) Viral loads were measured from plasma at the indicated time points after inoculation. The assay limit of detection is 10^2. (**c**) CD4 counts from peripheral blood were measured at the indicated time points after inoculation. Good and poor neutralizer animals, as determined by plasma neutralization, are shown in red and blue, respectively.

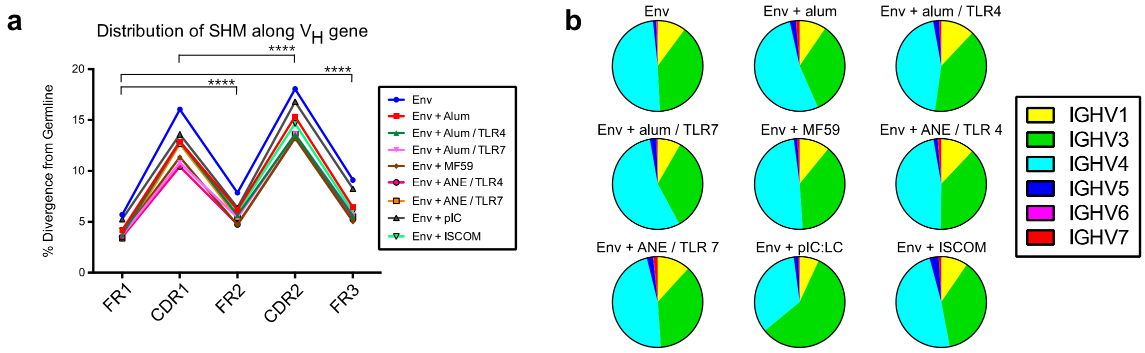

Supplementary Figure 7. Vaccine deep sequencing dataset overview. (**a**) Distribution of SHM along the V_H gene. Percent divergence from germline was plotted for the framework (FR) or complementarity determining regions (CDRs), averaged for each vaccine. CDR3 divergence cannot be accurately determined because of *N*- addition. Statistics are derived from 2-way ANOVA with Bonferroni correction. (**b**) V_H family composition separated by vaccine; the frequency of reads mapping to each V_H gene are represented as a fraction of the total sequences.

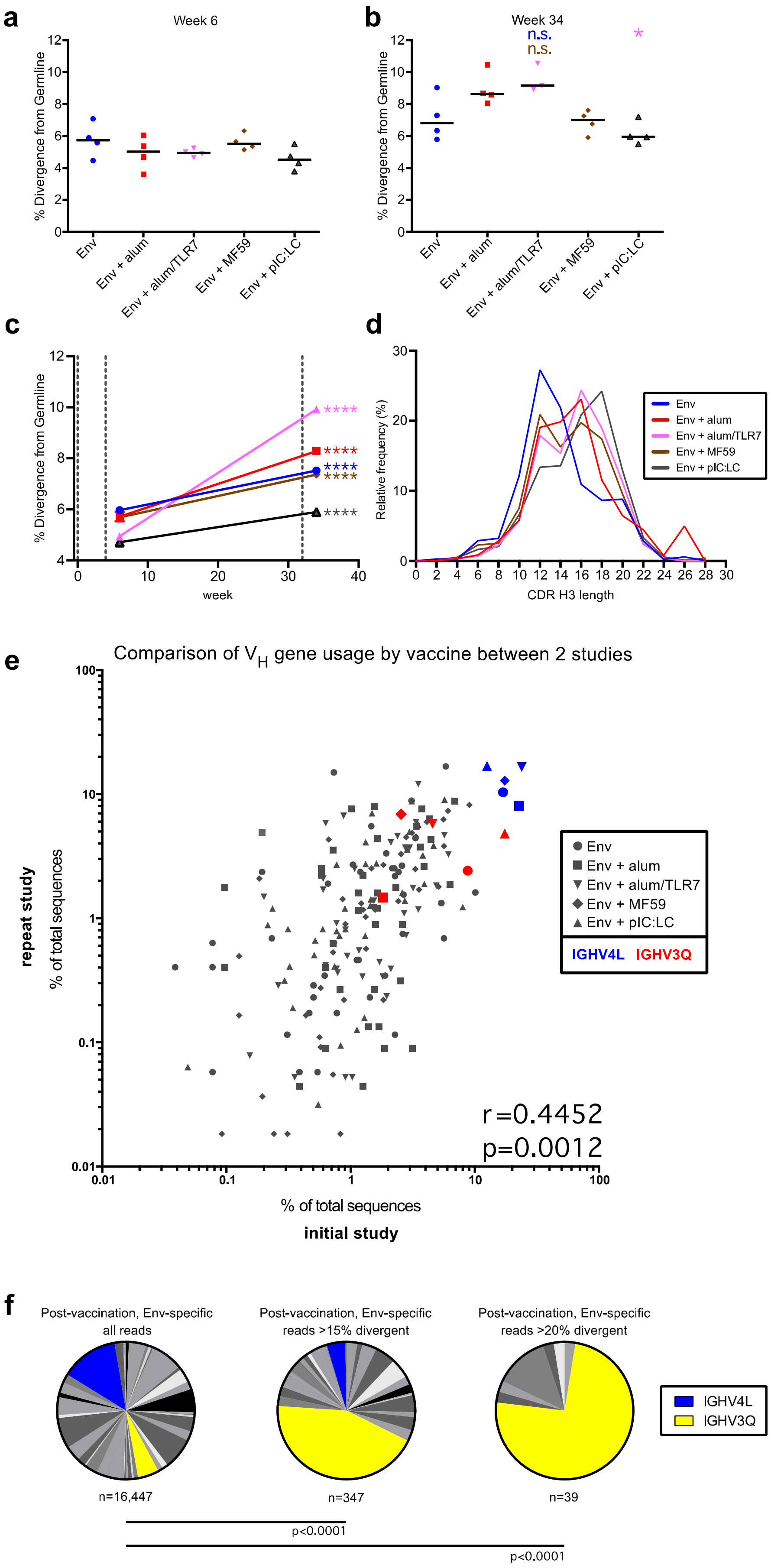
a
Week 6
% Divergence from Germline
Env
Env + alum
Env + alum/TLR7
Env + MF59
Env + pIC:LC
b
Week 34
n.s.
n.s.
*
c
% Divergence from Germline
week
****
d
Relative frequency (%)
CDR H3 length
Env
Env + alum
Env + alum/TLR7
Env + MF59
Env + pIC:LC
e
Comparison of V_H gene usage by vaccine between 2 studies
repeat study
% of total sequences
% of total sequences
initial study
IGHV4L
IGHV3Q
r=0.4452
p=0.0012
f
Post-vaccination, Env-specific all reads
n=16,447
Post-vaccination, Env-specific reads >15% divergent
n=347
Post-vaccination, Env-specific reads >20% divergent
n=39
IGHV4L
IGHV3Q
p<0.0001
p<0.0001

Supplementary Figure 8. Next-generation sequencing of antigen-specific B cells from a repeat study of protein and adjuvant vaccination. 24 experimentally-naïve NHP were immunized (n=4/group) with Env, Env+alum, Env+alum/TLR7, Env+MF59, Env+pIC:LC at weeks 0, 4 and 32. This study was undertaken to repeat the main protein/adjuvant vaccination study described in the material and methods section. (**a,b**) SHM for each animal (n=24) two weeks after the second immunization (week 6, **a**) or after the third immunization (week 34, **b**); each symbol represents the average percent divergence from germline for sequences from a given animal. Horizontal bars indicate medians. *, p<0.05 compared to the alum/TLR7 group by the Kruskal-Wallis test with a Dunn's correction. (**c**) Effect of boosting on SHM; data from weeks 6 and 34. Each symbol represents the mean percent divergence from germline from sequences from animals immunized with a given vaccine. Vertical dashed lines indicate time of immunizations. ****, p<0.0001 by two-way ANOVA test with Bonferoni correction. (**d**) CDR H3 length distribution as a composite from all sequences from a given vaccine; binning averaged in 2 aa increments. (**e**) V_H gene usage by vaccine: a comparison of initial and repeat Env/adjuvant vaccination studies. Sequences from the initial or repeat protein/adjuvant vaccine studies were first divided by vaccine. For each vaccine, the composition of individual V_H genes was determined as the percentage of sequences mapping to each V_H gene. Each symbol represents an individual V_H gene for each of five vaccine formulations. r- and p-values are from a Pearson correlation analysis between the initial and repeat studies showing that the distribution of V_H genes was highly reproducible between studies. Two V_H genes have been highlighted: IGHV4L (blue) shows an example of a V_H gene that is more uniformly used by each of the 5 vaccines; i.e. the adjuvants have little effect on IGHV4L composition. In contrast, IGHV3Q (red) shows a splay among the vaccines. The Env+alum formulation consistently induced fewer sequences mapping to IGHV3Q than did Env+alum/TLR7 or Env+pIC:LC. (**f**) V_H gene composition for all Env-specific sequences (left chart), sequences >15% divergent from germline (middle chart), or sequences >20% divergent from germline (right chart) from Env-specific B cells sorted from all animals post-vaccination. p values are derived from the Fisher's exact test for the proportion of IGHV3Q. The number of sequences in each dataset is indicated under the corresponding pie chart. Sequences mapping to IGHV4L (blue) and IGHV3Q (yellow) are highlighted.

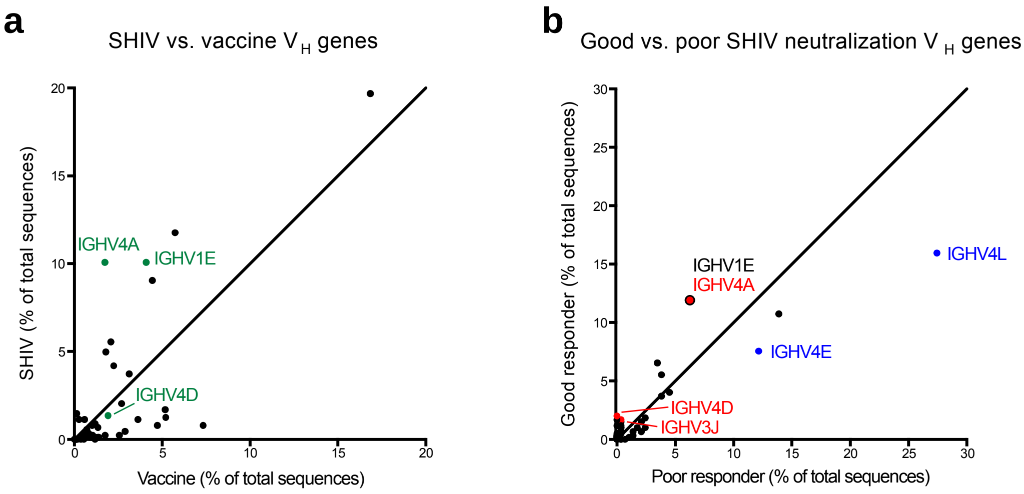

Supplementary Figure 9. V_H gene correlations between vaccination and an alternate $SHIV_{AD8}$ infection dataset. Here V_H gene composition data were collected after Sanger sequencing of single cell sorted Env-specific memory B cells collected 40 weeks post $SHIV_{AD8}$ infection using a YU2 gp140 probe[1]. This dataset was notably smaller than the deep sequencing dataset, containing only 884 unique sequences. The composition of individual V_H genes is graphed as the percentage of sequences mapping to each V_H gene within each dataset. Each dot represents an individual V_H gene; diagonal lines indicate the position of genes lacking a preference between a given comparison. (**a**) Env-specific sequences from this alternate $SHIV_{AD8}$ dataset compared to the Env vaccination dataset. V_H genes shown in Figure 6 to be enriched in composition in the $SHIV_{AD8}$ dataset are shown here in green. IGHV1E ($p<0.0001$, chi-square test) and 4A ($p<0.0001$, chi-square test) were enriched in these animals when compared to the vaccine study. (**b**) Env-specific sequences from $SHIV_{AD8}$ good neutralizers compared to poor neutralizers. V_H genes shown in Figure 6 to be enriched in composition in the good neutralizer dataset are shown here in red, and in the poor neutralizer dataset shown in blue. IGHV4A ($p=0.009$, Fisher's exact test) and 4D ($p=0.012$, Fisher's exact test) were confirmed as being enriched in good neutralizers with 3J ($p=0.115$, Fisher's exact test) also trending in the good neutralizers. IGHV4E ($p=0.033$, Fisher's exact test) and 4L ($p<0.0001$, Fisher's exact test) were confirmed as being enriched in poor neutralizers.

[1]Scheid, J.F., *et al.* A method for identification of HIV gp140 binding memory B cells in human blood. *Journal of immunological methods* **343**, 65-67 (2009).

Gene and allele name	Accession code	IMGT identifier (where applicable)
IGHV1-A*01	KP710506	
IGHV1-A*02	KP710507	
IGHV1-A*03	KP710508	
IGHV1-C*01	KP710512	
IGHV1-D*01	KP710531	
IGHV1-D*02	KP710509	
IGHV1-E*01	KP710532	
IGHV1-E*02	KP710533	
IGHV1-E*03	KP710534	
IGHV1-F*01	KP710535	
IGHV1-F*02	KP710536	
IGHV1-F*03	KP710510	
IGHV1-G*01	KP710537	
IGHV1-H*01	KP710511	
IGHV1-I*01	KP710581	
IGHV1-J*01	KP710538	
IGHV1-J*02	KP710539	
IGHV2-A*01	NW_001121240	IMGT_IGHV2-1*01
IGHV2-A*02	KP710540	
IGHV2-A*03	KP710541	
IGHV2-B*01	KP710542	
IGHV2-C*01	KP710543	
IGHV2-C*02	NW_001121239	IMGT_IGHV2-2*01
IGHV3-C*01	KP710544	
IGHV3-A*01	NW_001121240	IMGT_IGHV3-9*01
IGHV3-B*01	NW_001121239	IMGT_IGHV3-22*01
IGHV3-D*01	NW_001121239	IMGT_IGHV3-14*01
IGHV3-E*01	NW_001121239	IMGT_IGHV3-17*01
IGHV3-F*01	KP710545	
IGHV3-G*01	KP710513	
IGHV3-H*01	KP710527	
IGHV3-H*02	KP710528	
IGHV3-I*01	KP710546	
IGHV3-J*01	KP710547	
IGHV3-J*02	KP710548	
IGHV3-K*01	KP710549	
IGHV3-K*02	KP710514	
IGHV3-L*01	KP710550	
IGHV3-L*02	KP710515	
IGHV3-M*01	KP710551	
IGHV3-N*01	KP710516	
IGHV3-O*01	NW_001121240	IMGT_IGHV3-11*01
IGHV3-P*01	NW_001121239	IMGT_IGHV3-21*01
IGHV3-P*02	KP710517	
IGHV3-Q*01	KP710582	
IGHV3-Q*02	KP710583	
IGHV3-Q*03	KP710518	
IGHV3-R*01	NW_001121240	IMGT_IGHV3-10*01
IGHV3-S*01	NW_001121239	IMGT_IGHV3-20*01
IGHV3-S*02	KP710519	
IGHV3-T*01	KP710530	
IGHV3-U*01	KP710552	
IGHV3-U*02	NW_001121240	IMGT_IGHV3-5*01
IGHV3-V*01	KP710553	
IGHV3-V*02	NW_001121239	IMGT_IGHV3-13*01
IGHV3-W*01	NW_001121239	IMGT_IGHV3-18*01
IGHV3-W*02	KP710520	
IGHV3-X*01	NW_001121240	IMGT_IGHV3-12*01
IGHV3-Y*01	KP710554	
IGHV3-Y*02	KP710555	
IGHV3-Z*01	KP710529	
IGHV3-AA*01	KP710556	
IGHV3-AA*02	NW_001121240	IMGT_IGHV3-6*01
IGHV3-AB*01	NW_001121240	IMGT_IGHV3-7*01
IGHV4-A*01	KP710557	
IGHV4-A*02	KP710558	
IGHV4-B*01	KP710559	
IGHV4-B*02	KP710560	
IGHV4-C*01	KP710561	
IGHV4-D*01	KP710562	
IGHV4-E*01	KP710563	
IGHV4-E*02	KP710564	
IGHV4-E*03	KP710565	
IGHV4-F*01	KP710566	
IGHV4-F*02	KP710567	
IGHV4-G*01	KP710568	
IGHV4-G*02	KP710569	
IGHV4-H*01	KP710570	
IGHV4-I*01	KP710571	
IGHV4-J*01	KP710572	
IGHV4-K*01	KP710573	
IGHV4-L*01	KP710526	
IGHV4-L*02	KP710574	
IGHV4-L*03	NW_001121240	IMGT IGHV4-2*01
IGHV5-A*01	NW_001121239	IMGT_IGHV5-2*01
IGHV5-A*02	KP710575	
IGHV5-A*03	KP710576	
IGHV5-A*04	KP710577	
IGHV5-B*01	KP710521	
IGHV5-B*02	KP710578	
IGHV5-C*01	KP710522	
IGHV5-C*02	NW_001121240	IMGT_IGHV5-1*01
IGHV6-A*01	KP710579	
IGHV6-A*02	KP710523	
IGHV7-A*01	KP710524	
IGHV7-A*02	NW_001121240	IMGT_IGHV7-1*01
IGHV7-A*03	KP710580	
IGHV7-B*01	KP710525	

Supplementary Table 1. GenBank accession codes for NHP reference database. Codes starting with “KP” are newly identified by this study. Codes starting with “NW” are from the previously published NHP IMGT database.

Animal ID	Weeks post infection	Sample sorted	Number of cells sorted	Number of reads (QC)	Number of reads (unique)	Mean divergence from germline (%)	Germline divergence S.D.	Mean CDRH3 length (aa)	CDRH3 length S.D.
DBJI	6	+	980	4362	132	4.57	3.84	16.54	4.96
DBJI	6	-	48000	120899	5701	5.54	6.18	13.9	3.84
DBJI	29	+	1000	938	62	12.4	7.18	14.71	3.77
DBJI	29	-	53000	134662	2447	11.98	6.63	13.75	3.87
DBJI	54	+	4500	4203	74	9.74	4.86	16.55	4.76
DBJI	54	-	177000	157468	42487	8.92	5.38	14.15	3.63
DBJI	99	+	1600	3724	53	13.33	5.78	16.49	5.31
DBJI	99	-	75000	119002	10134	12.11	6.2	13.96	3.8
DBVC	6	+	220	2031	31	7.37	5.47	13.68	3.38
DBVC	6	-	27000	186609	1395	8.8	6.43	13.51	3.67
DBVC	99	-	13300	3278	213	11.27	6.48	13.63	3.29
DBVC	100	+	250	92	39	12.17	4.44	15.97	3.84
DBVC	100	-	20000	10445	2840	12.41	6.16	13.69	3.65
DBVC	110	+	113	4954	64	15.92	5.56	14.31	3.57
DBVC	110	-	1778	241851	1203	14.53	6.26	13.89	3.72
DBZ3	7	+	158	716	31	5.28	3.54	17.1	6.12
DBZ3	7	-	47000	2257	606	8.36	5.96	14.02	3.46
DBZ3	58	+	1052	3839	121	11.83	5.09	15.63	4.65
DBZ3	58	-	42000	240690	3612	10.4	6.1	13.88	3.56
DBZ3	91	+	1475	71700	817	14.56	4.52	16.26	4.86
DBZ3	91	-	5441	171938	1520	11.61	5.92	14.26	3.52
DBZ3	99	+	1160	818	200	14.15	4.54	16.25	5.13
DBZ3	99	-	40000	57239	10585	10.03	6.09	14.27	3.38
DC6W	8	+	135	306	13	13.2	6.07	15	6.71
DC6W	8	-	12500	97887	556	12.01	6.49	13.06	3.91
DC6W	32	+	850	7824	222	10.15	5.32	13.57	3.95
DC6W	55	-	2580	99180	786	11.69	6.19	13.84	3.87
DC6W	98	+	320	7858	228	19.16	5.99	13.12	4.21
DC6W	98	-	10700	33148	209	12.77	7.76	14.93	3.84
DC8T	6	+	52	522	19	5.02	5.47	13.42	4.02
DC8T	6	-	30600	116251	9999	8.61	6.05	13.8	3.56
DC8T	28	+	3500	6660	614	10.15	3.9	16.89	5.05
DC8T	28	-	92300	120117	27018	8.24	5.44	14.18	3.48
DC8T	52	+	1240	824	113	12.93	4.71	17.31	5.29
DC8T	52	-	42000	111186	9302	9.06	6.05	14.33	3.71
DC8T	98	+	500	89702	399	12.94	6.33	12.99	4.07
DC8T	98	-	25000	153620	3677	11.76	6.81	14.04	3.8
DCC7	6	+	206	2744	43	6.48	5.34	12.37	3.96
DCC7	6	-	37500	73996	5126	8.4	6.01	13.28	3.62
DCC7	29	+	218	5930	29	11.67	4.47	16.34	4.49
DCC7	29	-	22500	498867	16538	7.07	5.15	14.37	3.62
DCC7	54	+	206	1498	8	14.78	4.94	17.75	1.04
DCC7	91	+	463	141	29	14.55	7.39	16.69	4.36
DCC7	91	-	57200	82789	1850	6.83	5.9	14.07	3.27
DCC7	117	-	5400	457286	3809	9.24	6.36	14.46	3.75
DCF1	6	+	306	4363	104	5.83	4.27	16.28	4.93
DCF1	6	-	20144	160578	6623	7.13	5.41	14.1	4.04
DCF1	28	+	850	3052	191	11.76	3.88	18.6	5.9
DCF1	28	-	46400	153746	32165	9.33	4.89	14.53	3.63
DCF1	52	+	1150	12029	418	14.15	4.01	17.21	6.43
DCF1	52	-	61500	299326	26137	10.23	5.43	14.57	3.62
DCF1	102	+	1635	288	157	16.01	3.63	15.36	4.49
DCF1	102	-	87800	123386	31338	10.72	6.02	14.64	3.56
DCF1	108	+	2007	38042	1161	17.39	3.88	16.76	6.32
DCF1	108	-	53179	172193	31543	11.62	5.68	14.87	3.5
DCV9	6	+	63	263	11	5.44	4.24	16.28	4.93
DCV9	6	-	19000	100352	1512	9.26	6.1	14.1	4.04
DCV9	28	+	1100	4625	54	10.89	4.64	17.63	5.63
DCV9	28	-	83000	109311	13861	10.53	5.59	14.32	3.79
DCV9	52	+	370	5096	68	11.05	5.24	16.66	5.81
DCV9	52	-	35000	148442	4297	11.24	5.9	14.34	3.78
DCV9	98	+	300	456	21	13.74	4.83	16.48	6.18
DCV9	98	-	18800	159007	2185	11.9	6.36	14.13	3.97

Supplementary Table 2. 454 sequencing and bioinformatic analysis of B cells from $SHIV_{AD8}$-infected NHPs

Animal ID	Vaccine	Number of cells sorted	Number of reads (QC)	Number of reads (unique)	Mean divergence from germline (%)	Germline divergence S.D.	Mean CDRH3 length (aa)	CDRH3 length S.D.
05C045	Env	655	19147	360	12.34	7.32	13.74	4.16
CK8W	Env	900	27515	805	17.06	7.19	14.71	4.02
DB7D	Env	2000	9883	270	13.8	6.58	14.13	3.99
DBC8	Env	150	2034	119	17.7	6.26	13.71	4.08
DBEN	Env	3000	19504	941	13.75	5.82	13.82	3.9
DBEV	Env	160	1336	91	12.68	6.78	13.87	3.34
DB5Y	Env + Alum	750	6939	184	10.15	5.43	13.74	3.96
DB7N	Env + Alum	1000	30798	334	12.44	4.85	14.05	3.58
DB8W	Env + Alum	600	1389	54	10.35	4.71	16.15	3.46
DBPK	Env + Alum	950	17362	280	11.03	4.74	14.82	3.61
DBWZ	Env + Alum	237	5838	158	9.97	5.08	14.62	4.73
O4E065	Env + Alum	3300	20215	1061	12.99	5.15	14.98	4.06
A2E028	Env + Alum / TLR 4	3500	37460	1390	10.51	5.16	14.78	3.84
CL3B	Env + Alum / TLR 4	1100	5886	277	12.23	6.11	15.03	3.17
DBA8	Env + Alum / TLR 4	3500	32497	1054	11.82	4.63	14.34	3.56
DBR8	Env + Alum / TLR 4	900	28447	387	9.89	4.94	14.58	3.51
O4E114	Env + Alum / TLR 4	7000	33726	1699	10.78	4.58	14.38	4.11
CK8V	Env + Alum / TLR 7	3200	33459	1078	12.48	5.76	15.16	3.68
CL47	Env + Alum / TLR 7	3100	19664	1077	11.04	4.92	13.72	3.58
DB7F	Env + Alum / TLR 7	3000	16988	983	10.98	6.74	13.93	3.87
DBX4	Env + Alum / TLR 7	1700	19078	735	9.77	4.84	14.54	3.55
LWK	Env + Alum / TLR 7	278	3032	137	12.11	5.24	14.62	4.18
O4E113	Env + Alum / TLR 7	9100	33553	2484	10.17	4.98	15.13	3.48
A3E028	Env + MF59	15500	40277	4222	10	4.23	14.91	3.57
A3E049	Env + MF59	557	1258	114	11.57	5.01	15.24	3.89
DB9F	Env + MF59	3073	20018	1007	10.58	4.18	14.42	3.44
DBAR	Env + MF59	9000	27100	1942	10.5	4.37	14.88	3.49
O4E107	Env + MF59	2500	14265	912	10.84	4.76	15.1	3.77
T5325	Env + MF59	1550	10467	511	11.91	5.11	15.13	3.86
A3E037	Env + ANE / TLR 4	2600	9734	994	9.4	5.31	14.77	3.86
A4E005	Env + ANE / TLR 4	5000	18343	1630	9.62	4.26	14.54	4.1
DBV7	Env + ANE / TLR 4	6000	19693	1818	10.58	5.29	14.67	3.94
O4E064	Env + ANE / TLR 4	6000	724	14	21.18	6.28	12.5	2.24
CM45	Env + ANE / TLR 7	550	5457	152	12.51	5.29	13.72	4.09
DB4L	Env + ANE / TLR 7	3000	30359	1900	10.76	4.71	15.07	3.72
DB86	Env + ANE / TLR 7	6500	23469	2591	11.46	4.9	14.19	3.59
DBC6	Env + ANE / TLR 7	10000	3654	143	10.16	5.24	14.93	3.29
O4E087	Env + ANE / TLR 7	13500	34522	2033	9.98	4.56	14.65	3.71
T5320	Env + ANE / TLR 7	11000	32734	1062	10.29	4.19	14.61	3.68
A4E001	Env + pIC:LC	2200	25501	2272	16.46	6.27	13.75	3.87
A4E002	Env + pIC:LC	15000	39864	3313	10.77	6.19	14.94	3.6
CL24	Env + pIC:LC	1420	22041	842	15.86	6.94	14.11	3.6
DB76	Env + pIC:LC	7500	34680	1492	10.6	5.39	14.72	3.7
DBF7	Env + pIC:LC	4000	24514	1422	11.56	5.91	13.8	3.75
O4E016	Env + pIC:LC	800	22858	878	16.92	6.73	13.96	4
CL85	Env + ISCOM	2500	21511	1898	11.44	5.62	14.67	3.43
DBE6	Env + ISCOM	1000	13476	244	18.77	7.44	13.34	4.08
DBEI	Env + ISCOM	8000	16777	1748	11.98	4.57	13.95	3.43
DBX1	Env + ISCOM	2000	16512	783	10.02	4.68	14.8	3.61
O4E070	Env + ISCOM	9000	20735	1663	11.12	5.51	14.27	3.71
T5321	Env + ISCOM	1400	242	5	17.81	5.8	11.8	2.68

Supplementary Table 3. 454 sequencing and bioinformatic analysis of B cells from Env-vaccinated NHPs

Group	Vaccine	Number of reads (QC)	Number of reads (unique)	Mean divergence from germline (%)	Germline divergence S.D.	Mean CDRH3 length (aa)	CDRH3 length S.D.
1	Env	79419	2586	14.74	6.87	14.11	3.99
2	Env + alum	82541	2071	12.08	5.18	14.7	3.99
3	Env + alum / TLR 4	138016	4807	10.94	4.93	14.54	3.82
4	Env + alum / TLR 7	125774	6494	10.82	5.47	14.64	3.66
5	Env + MF59	113385	8708	10.40	4.41	14.88	3.58
6	Env + ANE / TLR 4	48494	4456	10.00	5.01	14.64	3.98
7	Env + ANE / TLR 7	130195	7881	10.75	4.73	14.58	3.68
8	Env + pIC:LC	169458	10219	13.07	6.75	14.33	3.77
9	Env + ISCOM	89253	6341	11.61	5.52	14.33	3.57

Supplementary Table 4. 454 sequencing and bioinformatic analysis of B cells by vaccine/adjuvant

Primer	Name	Sequence (5'-->3')
	**Multiplex Primer PCR**	
5'	XLR-B_VH1 LEADER-A	CCTATCCCCTGTGTGCCTTGGCAGTCTCAG ATGGACTGGACCTGGAGGAT
5'	XLR-B_VH1 LEADER-B	CCTATCCCCTGTGTGCCTTGGCAGTCTCAG ATGGACTGGACCTGGAGCAT
5'	XLR-B_VH1 LEADER-C	CCTATCCCCTGTGTGCCTTGGCAGTCTCAG ATGGACTGGACCTGGAGAAT
5'	XLR-B_VH1 LEADER-D	CCTATCCCCTGTGTGCCTTGGCAGTCTCAG GGTTCCTCTTTGTGGTGGC
5'	XLR-B_VH1 LEADER-E	CCTATCCCCTGTGTGCCTTGGCAGTCTCAG ATGGACTGGACCTGGAGGGT
5'	XLR-B_VH1-LEADER-F	CCTATCCCCTGTGTGCCTTGGCAGTCTCAG ATGGACTGGATTTGGAGGAT
5'	XLR-B_VH1-LEADER-G	CCTATCCCCTGTGTGCCTTGGCAGTCTCAG AGGTTCCTCTTTGTGGTGGCAG
5'	XLR-B_VH3 LEADER-A	CCTATCCCCTGTGTGCCTTGGCAGTCTCAG TAAAAGGTGTCCAGTGT
5'	XLR-B_VH3 LEADER-B	CCTATCCCCTGTGTGCCTTGGCAGTCTCAG TAAGAGGTGTCCAGTGT
5'	XLR-B_VH3 LEADER-C	CCTATCCCCTGTGTGCCTTGGCAGTCTCAG TAGAAGGTGTCCAGTGT
5'	XLR-B_VH3 LEADER-D	CCTATCCCCTGTGTGCCTTGGCAGTCTCAG GCTATTTTTAAAGGTGTCCAGTGT
5'	XLR-B_VH3 LEADER-E	CCTATCCCCTGTGTGCCTTGGCAGTCTCAG TACAAGGTGTCCAGTGT
5'	XLR-B_VH3 LEADER-F	CCTATCCCCTGTGTGCCTTGGCAGTCTCAG TTAAAGCTGTCCAGTGT
5'	XLR-B_VH4 LEADER-A	CCTATCCCCTGTGTGCCTTGGCAGTCTCAG ATGAAACACCTGTGGTTCTTCC
5'	XLR-B_VH4 LEADER-B	CCTATCCCCTGTGTGCCTTGGCAGTCTCAG ATGAAACACCTGTGGTTCTT
5'	XLR-B_VH4 LEADER-C	CCTATCCCCTGTGTGCCTTGGCAGTCTCAG ATGAAGCACCTGTGGTTCTT
5'	XLR-B_VH4 LEADER-D	CCTATCCCCTGTGTGCCTTGGCAGTCTCAG ATGAAACATCTGTGGTTCTT
5'	XLR-B_VH5 LEADER-A	CCTATCCCCTGTGTGCCTTGGCAGTCTCAG TTCTCCAAGGAGTCTGT
5'	XLR-B_VH5 LEADER-B	CCTATCCCCTGTGTGCCTTGGCAGTCTCAG CCTCCACAGTGAGAGTCTG
5'	XLR-B_VH6 LEADER-A	CCTATCCCCTGTGTGCCTTGGCAGTCTCAG ATGTCTGTCTCCTTCCTCATC
5'	XLR-B_VH7 LEADER-A	CCTATCCCCTGTGTGCCTTGGCAGTCTCAG GGCAGCAGCAACAGGTGCCCA
3'	XLR-A_**BARCODE**_IgG	CCATCTCATCCCTGCGTGTCTCCGACTCAG **XXXXX** GGGGAAGACCGATGGGCCCTTGGT
	**RACE PCR**	
5' rxn A	SmarterOligo	AAGCAGTGGTATCAACGCAGAGTACATrGrGrG *rG=riboguanosine
3' rxn A	IgG	GCCAGGGGGAAGACCGATGGGCCCTTGGTGGA
3' rxn A	IgM	GAGACGAGGGGGAAAAGGGTTGGGGCGGATGCA
5' rxn B	XLR-A_SmarterOligo	CCATCTCATCCCTGCGTGTCTCCGACTCAG AAGCAGTGGTATCAACGCAGAGT
3' rxn B	XLR-B_IgG	CCTATCCCCTGTGTGCCTTGGCAGTCTCAG GGAAGACCGATGGGCCCTTGGTGG
3' rxn B	XLR-B_IgM	CCTATCCCCTGTGTGCCTTGGCAGTCTCAG GAGGGGAAAAGGGGTTGGGGCGG

Supplementary Table 5. Primers for multiplex and RACE PCR
